# The titin N2A-MARP signalosome constrains muscle longitudinal hypertrophy in response to stretch

**DOI:** 10.7554/eLife.107597

**Published:** 2026-07-30

**Authors:** Robbert van der Pijl, Jochen Gohlke, Joshua Strom, Eva Peters, Shengyi Shen, Stefan Conijn, Zaynab Hourani, Stephan Lange, Ju Chen, Paul Langlais, Siegfried Labeit, Henk L Granzier, Coen Ottenheijm

**Affiliations:** 1 Department of Cellular and Molecular Medicine, University of Arizona Tucson United States; 2 Department of Physiology, Amsterdam University Medical Center, VUMC Amsterdam Netherlands; 3 School of Medicine, University of California San Diego San Diego United States; 4 Department of Biomedicine, Aarhus University Aarhus Denmark; 5 Department of Endocrinology, University of Arizona Tucson United States; 6 Department of Integrative Pathophysiology, Medical Faculty Mannheim Mannheim Germany; The University of Melbourne Australia; Universitat Pompeu Fabra Spain

**Keywords:** titin, MARP, hypertrophy, muscle, signaling, mechanosensing, Mouse, Rat

## Abstract

Titin-based mechanosensing is a key driver of trophic signaling in muscle, yet the downstream pathways linking titin sensing to muscle remodeling remain poorly understood. To investigate these signaling mechanisms, we utilized unilateral diaphragm denervation (UDD), an in vivo model that induces titin-stiffness-dependent hypertrophy via mechanical stretch. Using UDD in rats and mice, we characterized the longitudinal hypertrophic response and distinguished stretch-induced signaling from denervation effects by performing global transcriptomic and proteomic analyses following UDD and bilateral diaphragm denervation (BDD) in rats. Our findings identified upregulation of titin-associated muscle ankyrin repeat proteins (MARPs). Subsequent phosphorylation enrichment mass spectrometry in mouse diaphragm highlighted the involvement of the N2A-element. UDD in MARP knockout (KO) mice resulted in enhanced longitudinal hypertrophy, with Western blot analysis revealing activation of the mTOR pathway. Furthermore, pharmacological inhibition of mTORC1 with rapamycin suppressed longitudinal hypertrophy, demonstrating that mTOR signaling regulates titin-mediated hypertrophic growth in a MARP-dependent manner. These findings establish MARPs as key modulators of titin-based mechanotransduction and highlight mTORC1 as a central regulator of longitudinal muscle hypertrophy.

## Introduction

Titin is a giant protein in striated muscle and forms an elastic filament in sarcomeres (Labeit and Kolmerer, 1995), the smallest contractile unit in muscle. One of the functions attributed to titin is to generate passive tension in muscle (Horowits et al., 1986; Swist et al., 2020), ensuring optimal overlap of the actin-based thin filaments and myosin-based thick filaments for muscle contraction. The elastic properties of titin combined with its arrangement in the sarcomeres, spanning the half-sarcomere, make titin an ideal stress sensor. The mechanosensory properties of titin have been extensively described (Ottenheijm et al., 2011; van der Pijl et al., 2019; Voelkel and Linke, 2011) and appear to localize to several signaling hotspots on titin.

In skeletal muscle, the N2A element is the prevalent titin region associated with hypertrophy signaling. The N2A element is known for its interaction with the muscle ankyrin repeat proteins (MARP1-3) (Miller et al., 2003; Wette et al., 2017) and calpain3 (Hayashi et al., 2008). All MARPs have been implicated in trophicity signaling, with redundancy between the MARP proteins (Miller et al., 2003; Lange et al., 2016). MARP1 tethers titin to the thin filament, forming a mechanism for increasing passive tension (van der Pijl et al., 2021b; Zhou et al., 2021). In the heart, MARP1 also interacts with MLP and protein kinase C alpha in intercalated disks, resulting in a maladaptive trophic response in dilated cardiomyopathy (Lange et al., 2016). MARP2 can be phosphorylated at serine-99 (S69 in mice) by Akt, resulting in reduced differentiation potential of myoblasts (Cenni et al., 2011). MARP2 has also been linked to the NFkB-pathway in inflammatory responses (Bean et al., 2014), suggesting MARP2 may have roles in atrophy signaling. MARP3 is the least studied member of the MARPs but appears to play a role in glucose uptake and vascular remodeling in skeletal muscle (Shimoda et al., 2015; Laughlin et al., 2017). Titin has previously been shown to activate signaling in response to stretch (Mayans et al., 1998; Bertz et al., 2009), providing a potential activation mechanism for N2A to activate trophic signaling.

Muscle hypertrophy is directly associated with titin-based stiffness. High stiffness, as observed in the Ttn^Δex112-158^ and Ttn^Δex219-225^ mouse models (van der Pijl et al., 2020; Brynnel et al., 2018), results in longitudinal hypertrophy, i.e., an increase in serial-linked sarcomeres, to reduce sarcomere length and normalize passive tension. We previously used a surgical model in which we denervated one hemi-diaphragm (unilateral diaphragm denervation, UDD) (van der Pijl et al., 2018), inducing passive cyclic stretch of the denervated hemi-diaphragm by the innervated costal to induce (longitudinal) hypertrophy. This hypertrophy was dependent on titin-based stiffness, as higher titin stiffness resulted in exaggerated hypertrophy and lower titin stiffness resulted in attenuated hypertrophy (van der Pijl et al., 2018). UDD is a unique model as it allows the in vivo study of passive stretch-induced muscle hypertrophy.

We used UDD as a model for studying titin-mechanosensing and examined the signaling that underlies the hypertrophy response. Our findings support the N2A-MARP signalosome inhibiting longitudinal hypertrophy and show mTOR signaling to be a prominent contributor to longitudinal hypertrophy.

## Results

### Longitudinal hypertrophy regulates transient trophicity in UDD

**Figure 1.**
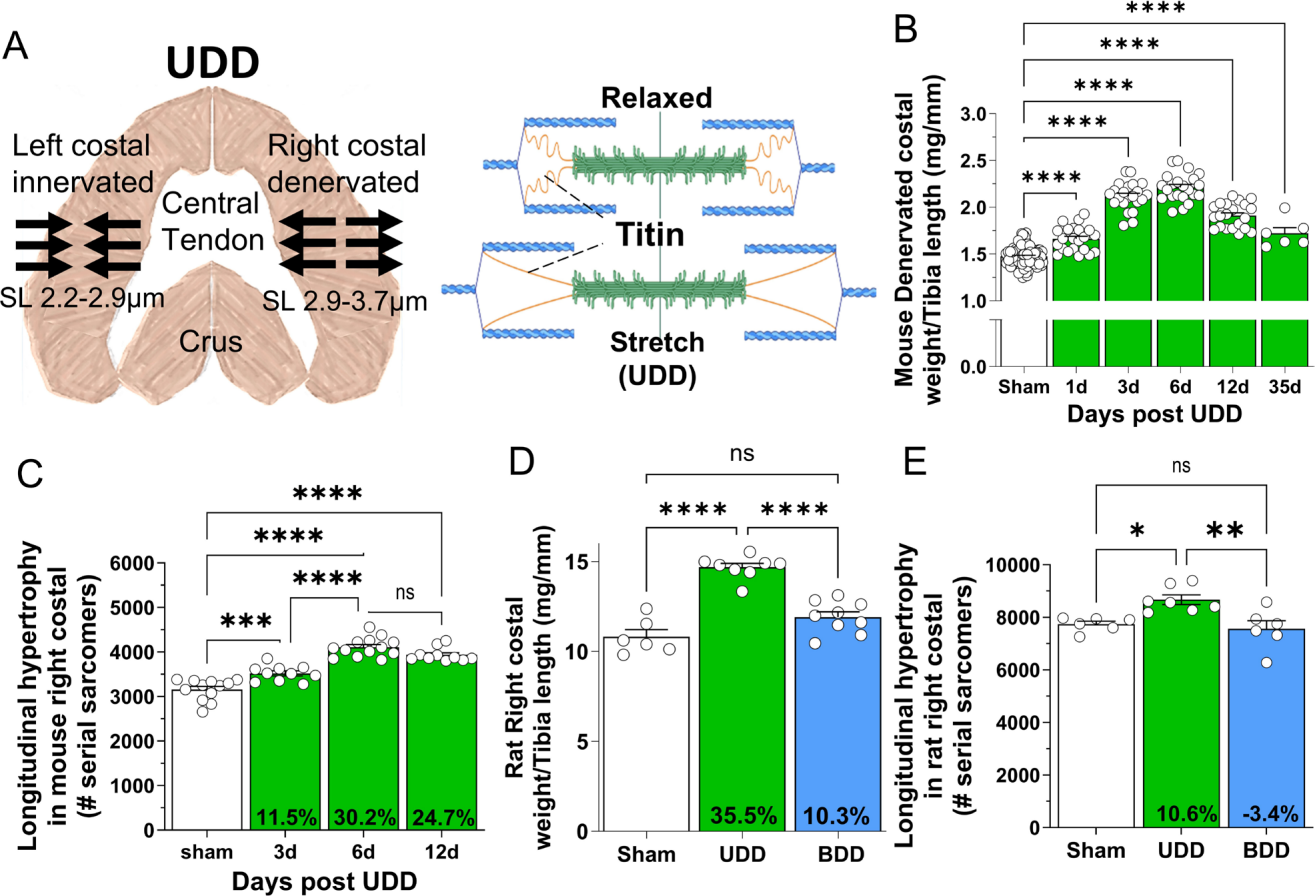
Transient hypertrophy following unilateral diaphragm denervation (UDD) in the denervated costal diaphragm. (**A**) Schematic of how UDD affects sarcomere length in the denervated costal (left panel) and how stretch extends titin for mechanosensing (right panel). (**B**) Mouse diaphragm costal weight normalized to tibial length following 1, 3, 6, 12, and 35 days of UDD, showing the hypertrophy phase peaking at 6 days and progressing to the atrophy phase at 12 days post-UDD (n=6–22, shams grouped for simplicity). A substantial part of the hypertrophy seen in mice encompasses longitudinal hypertrophy (**C**), lengthening of the muscle fibers by addition of serial sarcomeres (n=10–13). The increased fiber length likely reduces the stretch-based hypertrophy signaling and thus explains the transient nature of hypertrophy. (**D**) Three-day bilateral diaphragm denervation (BDD) in rats (n=6–9) confirms stretch is the trigger for inducing hypertrophy in UDD at the tissue mass level (D; rat diaphragm right costal normalized to tibial length, denervated in UDD) and serial sarcomeres level (**E**). One-way ANOVA, with Tukey post-hoc testing. Bar graphs denote mean ± SEM. Figure 1A was created with BioRender.com.

A unique aspect of unilateral diaphragm denervation (UDD) is the transient nature of the hypertrophy. This transient nature is likely the result of changes in fractional extension of sarcomeres. Sarcomere length (SL) at end-expiration length was 2.9±0.1 µm in sham, while stretch lengths at end-inspiration were predicted to reach 3.7 µm directly after denervation of the right costal diaphragm (Figure 1A, previously published van der Pijl et al., 2018). Such sarcomere lengths put high strain on titin (Figure 1A, right panel), providing a potent trigger for mechanosensing and subsequent hypertrophy signaling. Our results show that during 6 days of UDD, the denervated costal rapidly hypertrophies, followed by slow onset of atrophy (Figure 1B). The mass increase coincides with longitudinal hypertrophy of muscle fibers, addition of serial-linked sarcomeres, adding 952±81 sarcomeres (30.2% increase in total fiber length; *p*<0.0001; Figure 1C) following 6 days of UDD. This increase in sarcomere number appeared to stabilize by 6 days of UDD, as at 12 day UDD sarcomere addition was measured to be 778±87 sarcomeres versus sham levels (not significantly different compared to 6 day UDD). Assuming that fractional extension of sarcomeres during inspiration decreases with longitudinal hypertrophy, 30.2% increase in sarcomere number would reduce the sarcomere length at end inspiration from 3.7 µm to ~2.8 µm, in close agreement with whole-body formaldehyde perfusion experiments which showed sarcomere lengths of 2.7±0.1 µm (van der Pijl et al., 2018). As a SL of 2.8 µm falls below the end-expiration SL, the denervated costal effectively no longer experiences ‘stretch,’ reducing titin’s mechanosensing trigger and thus explaining the transient nature of the hypertrophy seen in UDD.

**Figure 1—figure supplement 1.**
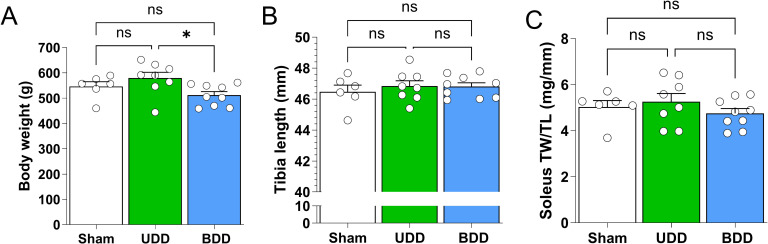
Three days bilateral diaphragm denervation in rats showed similar body weights compared to sham animals (A; n=6–9/group) and were of similar size based on tibia length (**B**) and soleus muscle weights (**C**). Statistical testing by one-way ANOVA and Dunnett’s multiple comparisons test. Bar graphs denote mean ± SEM.

**Figure 1—figure supplement 2.**
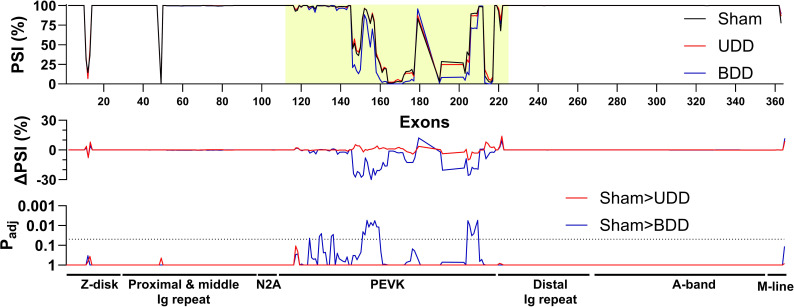
Titin splicing in 3 day unilateral and bilateral diaphragm denervation in rats showed similar levels of splicing between unilateral diaphragm denervation (UDD) (red line) and sham (black line) animals, while bilateral diaphragm denervation (BDD) (blue line) animals showed a decrease in exons coding for the elastic PEVK element. Statistical testing by Multiple t-testing, comparing sham to UDD and sham to BDD (n=5/group). Bar graphs denote mean ± SEM.

**Figure 1—figure supplement 3.**
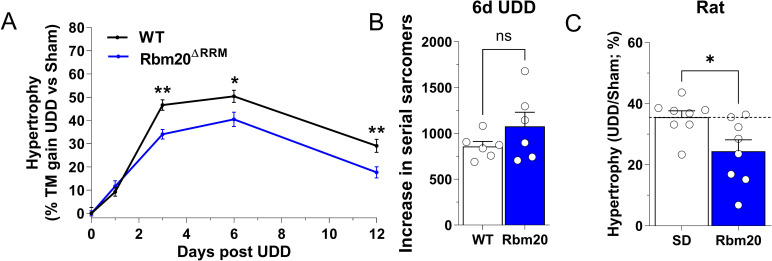
Role of titin stiffness on hypertrophy following unilateral diaphragm denervation (UDD). (**A**) Transient hypertrophy response in Rbm20^ΔRRM^ mice (more compliant titin) showing a blunted hypertrophy response compared to wild-type (WT) mice, based on percent increase of diaphragm right costal mass relative to sham (n=10–12). (**B**) Titin-based stiffness does not alter longitudinal hypertrophy response, as both WT and Rbm20^ΔRRM^ mice show a similar increase in serial sarcomeres following 6 days UDD. Rbm20 knockout (KO) rat response to 3 days UDD, based on percent increase of diaphragm right costal mass relative to sham (mouse n=10–11, rat n=8) supporting titin-based stiffness regulating muscle hypertrophy similarly across species. Statistical testing by t-test. Bar graphs denote mean ± SEM.

To confirm that stretch is the trigger for hypertrophy, rather than denervation, we performed bilateral diaphragm denervation in rats (BDD), resulting in complete inactivity of the diaphragm. Note that we attempted BDD in mice but experienced high mortality rates, while survival in rat was >75%. All rats were of similar body weight pre-surgery and showed a slight decrease in body weight after 3 day BDD (Figure 1—figure supplement 1; *p*=0.036), and comparable tibial length and soleus weights (Figure 1—figure supplement 1B and C). Similar to mice, rats present with increased denervated costal mass after 3 day UDD: 14.68±0.64 mg/mm versus sham rats 10.82±0.39 mg/mm (Figure 1D; *p*>0.0001; muscle weight normalized to tibial length). However, 3 day BDD rats did not develop increased mass 11.92±0.89 mg/mm compared to sham rats 10.82±0.39 mg/mm. The increase in costal mass at 3 day UDD results in part from an increase in longitudinal hypertrophy, with denervated costal diaphragm adding 933±301 serial sarcomeres compared to sham animals (Figure 1E; *p*=0.018), whereas 3 day BDD rats showed no difference in serial sarcomere number (7559±313 versus sham 7740±111). Thus, stretch and not denervation triggers longitudinal hypertrophy.

To support titin’s role as a mechanosensor of stretch to drive hypertrophy, we performed RNA-seq on both UDD and BDD operated rat diaphragms. Splicing appears mostly unaffected in UDD animals, while BDD rats showed marked increased splicing in exons encoding the elastic PEVK region of titin suggesting reduced titin compliance (Figure 1—figure supplement 2). To study how titin compliance might affect the hypertrophy response in UDD, we performed UDD on Rbm20^ΔRRM^ mice and Rbm20 KO rats. Rbm20 is an RNA splicing factor for titin and both the mouse and rat model generate larger, more compliant titin isoforms and should thus be less sensitive to stretch. Rbm20^ΔRRM^ mice showed relatively less tissue mass increase compared to wild-type (WT) 3, 6, or 12 days UDD (Figure 1—figure supplement 3A), without changes in longitudinal hypertrophy at 6 days UDD (Figure 1—figure supplement 3B) suggesting the difference in mass is a result from radial fiber growth, in agreement with our previous findings (van der Pijl et al., 2018). Finally, we compared denervated costal mass from Sprague Dawley (SD) and Rbm20 ko rats 3 day UDD, showing a difference in mass increase of 24.4±3.8% in Rbm20 KO and 35.5±5.9% in SD rats (*p*=0.02; Figure 1—figure supplement 3C), supporting that stretch evokes hypertrophy in muscle and that titin compliance modulates the extent of hypertrophy across species. Thus, muscle stretch in the costal diaphragm is a potent trigger for longitudinal muscle hypertrophy, with titin being a primary sensor.

### Global transcriptome and proteome level studies reveal a distinct subset of genes and proteins involved in UDD stretch hypertrophy

**Figure 2.**
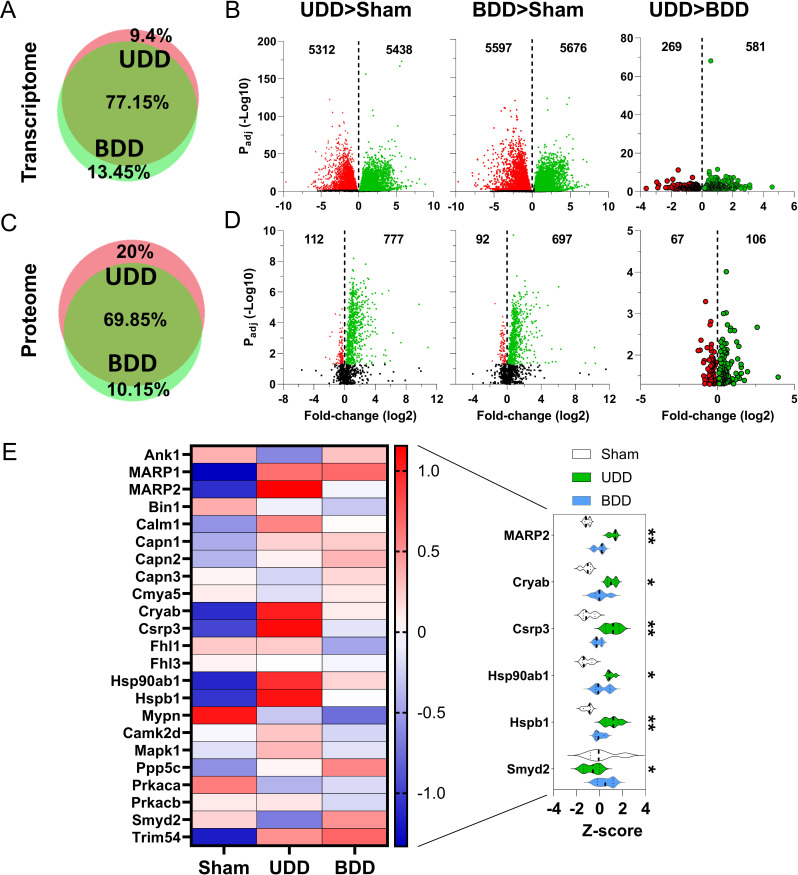
Global transcriptomics and proteomics following 3 days unilateral diaphragm denervation (UDD) and bilateral diaphragm denervation (BDD) in rats. Global transcript studies by RNA-seq of sham, UDD, and BDD right costal diaphragm (n=5/group). Same parameters apply to the global proteome studies (**C–D**) with mass spectrometry. Quantitative Venn diagrams of the transcriptome (**A**) showing overlap gene regulation between UDD and BDD. Vulcano plots of UDD (B, left) and BDD (B, middle) showed similar gene regulation. Comparing UDD to BDD directly revealed just 850 differentially regulated genes (B, right) indicating a small subset being responsible for hypertrophy regulation. Quantitative Venn diagrams of the proteome (**C**) showed similar regulation compared to transcriptome. Volcano plots of UDD (D, left) and BDD (D, middle) showed primarily upregulation of proteins. Comparing UDD to BDD directly revealed just 173 differentially regulated proteins (D, right). Green-dots: upregulated genes/proteins, red-dots: downregulated genes/proteins. Titin-associated proteins in heatmap of proteome (E; Z-score: red = upregulated, blue = downregulated) and violin plots (right panel) of differential proteins between UDD (red) and BDD (blue), indicating upregulation of titin-associate proteins following stretch. Two-way ANOVA (sham vs UDD and sham vs BDD) p_int_: *p<0.05, **p<0.01. Violin plot denotes median ± quartiles.

To identify the cellular processes involved in stretch-based hypertrophy, we performed both transcriptome-wide studies by RNA sequencing (RNA-seq) as well as proteome studies by global mass spectrometry (MS) on our rat 3 day sham, UDD, and BDD samples. Initial studies focused on separating the effect of denervation versus stretch. Principal component analysis of the top 500 genes showed close clustering of samples by group (Figure 2—figure supplement 1A), suggesting distinct gene programs are active between sham, UDD and BDD. At the protein level, principal component analysis of the top 250 proteins suggests narrow separation of groups, predicting overlap of the BDD and UDD samples (Figure 2—figure supplement 1B). Gene expression studies revealed very close overlap of BDD and UDD profiles with 77.15% overlap in differentially expressed genes (DEGs: p_adj_ <0.05) and just 9.4% of DEGs being specific for UDD (Figure 2A). Volcano plots of UDD >Sham (Figure 2B, left panel) and BDD >Sham (Figure 2B; middle panel) showed the ~11,000 DEGs (Supplementary file 1A-F) to be nearly equally distributed between up and downregulated DEGs. Direct comparison of UDD >BDD (Figure 2B, right panel) revealed 850 DEGs to be uniquely associated with UDD, of which 581 were upregulated (Supplementary file 1). MS studies broadly agreed with the RNA-seq data, showing UDD and BDD share overlapping expression programs (Figure 2A & C). Twenty percent of the differentially expressed proteins (DEPs; *p*<0.05) detected are unique to UDD (Figure 2C). Volcano plots of UDD >Sham (Figure 2D, left panel) and BDD >Sham (Figure 2D, middle panel) showed 889 and 789 DEPs, respectively, (Supplementary file 2A-F) which were primarily increased. Comparing UDD >BDD revealed 173 DEPs (Figure 2D, right panel) that are unique to UDD. Most of DEPs (and DEGs) found are related to transcription, translation, energetics, and folding, with the highest fold DEP’s in rat UDD >BDD being: *Med15* (15.5-fold), *Eif3i* (sixfold) and *Gtpbp3* (3.9-fold) (Supplementary file 2). GOterm enrichment of UDD >BDD, separated by downregulated and upregulated DEGs (Figure 1—figure supplement 3C, left top and bottom graph, respectively), and DEPs (Figure 2—figure supplement 1C, right top and bottom graph, respectively), indicated that upregulated DEG/DEPs are primarily related to muscle development and function consistent with an active hypertrophy program and downregulated targets with metabolism and cellular respiration. Concurrently, KEGG PATHWAY database searches (Supplementary file 1) indicated that the DEGs are involved in muscle remodeling. Thus, UDD has an expression profile that is distinct from denervation (BDD), with more focus on remodeling of muscle.

**Figure 2—figure supplement 1.**
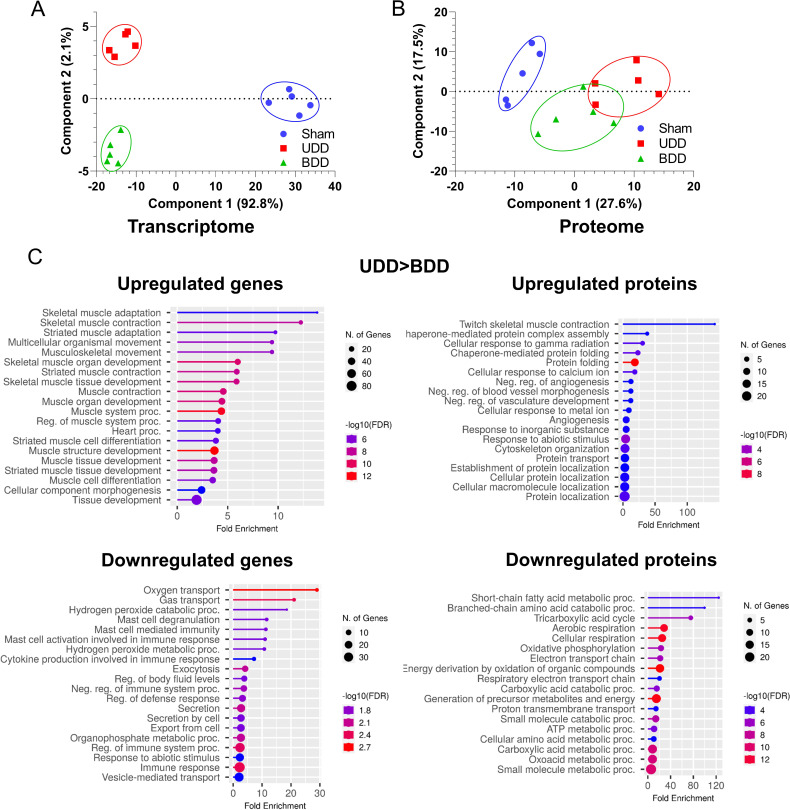
Principal component analysis of the rat 3 day unilateral diaphragm denervation (UDD) and bilateral diaphragm denervation (BDD) transcriptome (**A**) and proteome (**B**), showing clear separation of groups (n=5/group) at the transcript level and overlap of BDD and UDD samples at the protein level. Gene ontology (GO) term enrichment of UDD>BDD separated by up- or down-regulated transcriptomes and proteome (C, left and right, respectively) show distinct, yet overlapping cellular processes. Global mass spectrometry was analyzed by ANOVA and corrected for multiple comparisons with false discovery rate with a cut-off at *p*<0.05.

**Figure 2—figure supplement 2.**
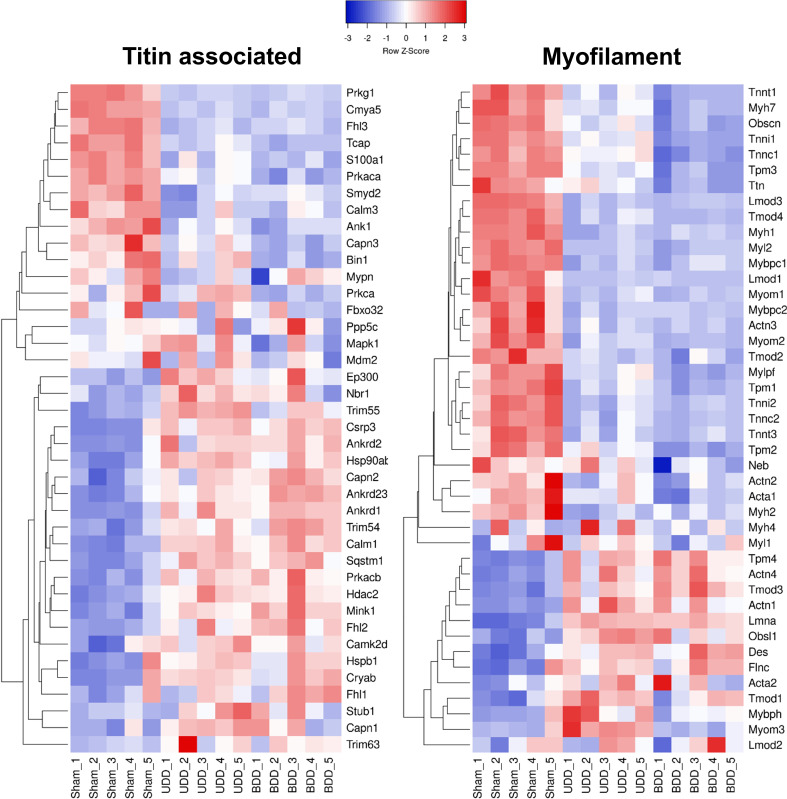
Transcriptome regulation of titin-associated and myofilament genes by RNA-seq in rats following 3 days of unilateral diaphragm denervation (UDD)/bilateral diaphragm denervation (BDD). Heatmaps showing similar regulation between UDD and BDD samples (n=5/group; Z-score: red = upregulated, blue = downregulated) at the transcript level for titin-associated and myofilament genes, based on hierarchical clustering.

Exploring if titin-mechanosensing was active in either UDD or BDD we generated heatmaps of titin-binding proteins based on RNA-seq (Figure 2—figure supplement 2) and global MS data (Figure 2E). To narrow down proteins that were differential between UDD and BDD we tested (two-way ANOVA p_int_ <0.1) the z-score and found: MARP2 (*Ankrd2*; *p*=0.003), Cryab (*p*=0.026), Csrp3 (*p*=0.002), Hsp90ab1 (*p*=0.09), Hspb1 (*p*=0.008). and Smyd2 (*p*=0.004) to be unique to UDD. Interestingly, these DEPs (MARP2, Smyd2, Hspb1, Hsp90ab and Cryab) are established binding partners of the N2A element of titin (van der Pijl et al., 2021a).

### Titin phosphorylation in UDD

**Figure 3.**
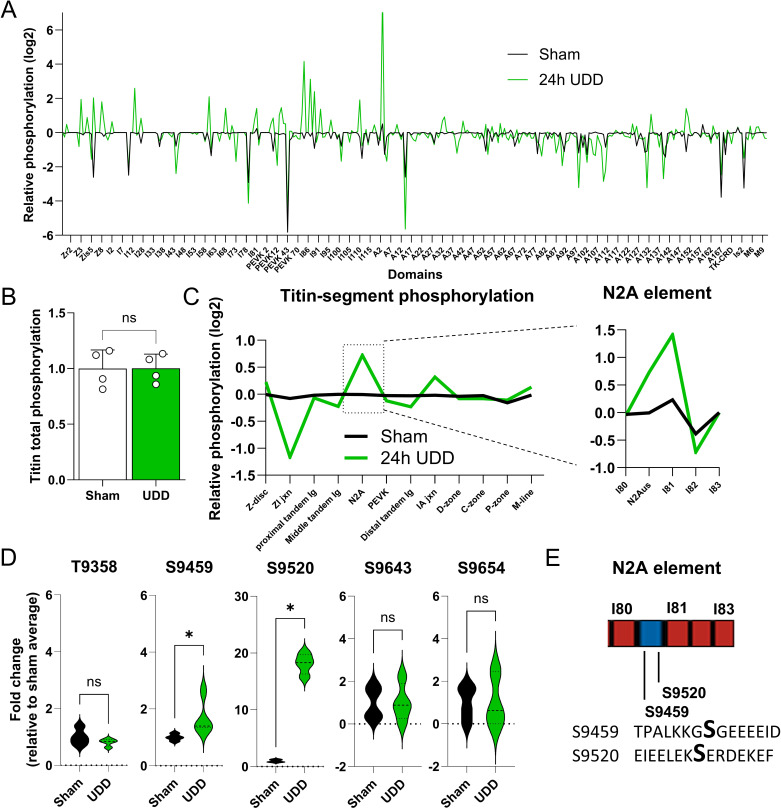
Phosphorylation of titin following 24 hr of unilateral diaphragm denervation (UDD) by mass spectrometry (n=4/group). Phosphorylation of individual titin domains (**A**) relative Z-score (log2) of titin phosphorylation showing domain specific changes in phosphorylation. Total phosphorylation of titin (**B**) is not affected by UDD, however, titin showed regional changes in phosphorylation (**C**), notably increased phosphorylation of the N2A-element (boxed). Fold-change of the phosphorylation signal for the five main sites found in the N2A-element (**D**). (**E**) Schematic of the N2A element with the 2 pSer found in the N2Aus (Transcript: ENSMUST00000099981.10 Ttn-203). Red: Ig domain coding and blue: unique sequence coding. Mouse titin phosphorylation and global mass spectrometry was analyzed by t-test, Kolmogorov-Smirnov test or multiple t-test with a cut-off at *p*<0.05. Bar graph denotes mean ± SEM and violin plots denote median ± quartiles.

With the rat data indicating changes in titin-associated signaling and titin stiffness modifying the hypertrophy response in mice, we evaluated how UDD affects titin post-translationally. We performed MS on phosphorylation-enriched peptides from 24 hr UDD diaphragm samples. 24 hr UDD was selected to study the early phosphorylation events. MS revealed 2870 phosphorylated peptides (*p*<0.05), of which 142-sites were in titin (~700-sites identified including non-significant sites; Supplementary file 3A). We opted to define titin phosphorylation by analyzing the phosphorylation at the domain level to study if titin showed regional changes in phosphorylation (Figure 3A and Supplementary file 3), as total titin phosphorylation (Figure 3B) was unchanged. Domain-level analysis indicated titin phosphorylation was primarily changing in the I-band. This motivated quantifying titin phosphorylation by segment (Figure 3C and Supplementary file 3; following established naming conventions in Labeit and Kolmerer, 1995, and Bang et al., 2001). Segmental analysis of titin (Figure 3C) revealed a marked increase in phosphorylation of the N2A-element and I/A-junction, and a decrease in the Z/I-junction. Focusing on the increase in phosphorylation of the N2A-element, a known trophicity signaling hub (reviewed in van der Pijl et al., 2021a), we assessed the specific domain phosphorylation of the N2A-element (Figure 3C, highlight). In the N2A-element, we found seven phosphorylation sites (Figure 3D–E) of which S9346 & S9350 are located in a linker sequence between I79 and I80, S9483 in I80, S9459, & S9520 in the N2A unique sequence, S9654 in I81 and S9643 in I82. The two sites located in the N2A unique sequence were significantly upregulated following UDD (Figure 3D–E). pS9459 and pS9520 currently have no known function but could serve as a recruitment signal (see discussion).

**Figure 3—figure supplement 1.**
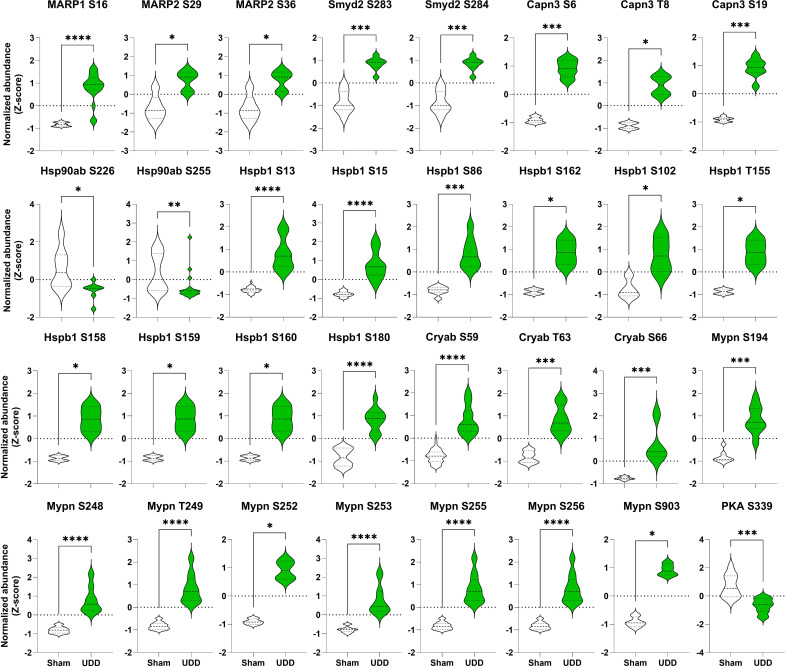
Titin N2A associated protein phosphorylation events at 24 hr unilateral diaphragm denervation (UDD). Violin plots of phosphorylation events in N2A-associated proteins following UDD: MARP1 (Transcript: ENSMUST00000237142.2 *Ankrd1-205*), MARP2 (Transcript: ENSMUST00000026172.3 *Ankrd2-201*), *Smyd2* (Transcript: ENSMUST00000027897.8 *Smyd2-201*), *Capn3* (Transcript: ENSMUST00000028749.15 *Capn3-202*), *Hsp90ab* (Transcript: ENSMUST00000024739.14 *Hsp90ab1-201*), *Mypn* (Transcript: ENSMUST00000095580.3 *Mypn-201*), *Hspb1* (Transcript: ENSMUST00000005077.7 *Hspb1-201*), *Cryab* (Transcript: ENSMUST00000217475.2 *Cryab-206*), and *Prkca*/PKA (Transcript: ENSMUST00000005606.8 *Prkaca-201*). Data n=4/group represented as Log2 of the normalized abundance with significance determined by Kolmogorov-Smirnov test. Violin plot denotes median ± quartiles.

### The MARP proteins regulate hypertrophy in UDD

**Figure 4.**
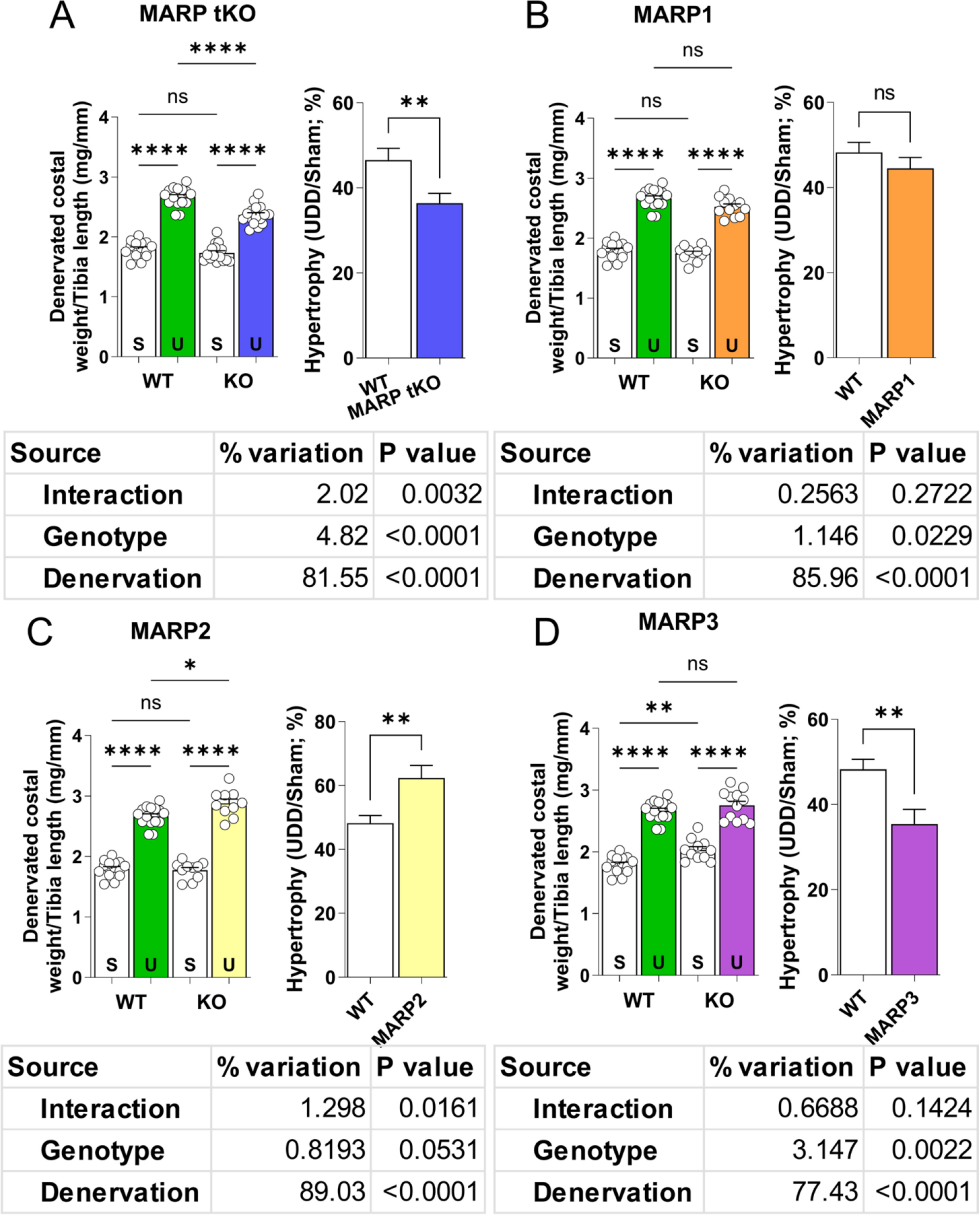
Six-day unilateral diaphragm denervation (UDD) on knockout (KO) mice of muscle ankyrin repeat proteins (MARPs). MARP triple knockouts (KOs) show a reduced response to UDD (A; *p*<0.01). No effect of single MARP1 KO (**B**) on UDD, increased hypertrophy following MARP2 KO (B; *p*<0.01; t-test), indicating possible roles in hypertrophy suppression or atrophy signaling and MARP3 KO (**C**) showed baseline hypertrophy in costal diaphragm in addition to less hypertrophy development in UDD (*p*<0.01; t-test) compared to wild-type (WT), implying roles as a suppressor of hypertrophy. Left panel, diaphragm right costal mass normalized to tibial length and right panel, percentual increase in right costal mass relative to sham. S=Sham, U=UDD (n=10–12). Statistical testing by t-test or two-way ANOVA with Tukey post-hoc testing. Bar graphs denote mean ± SEM.

The MARP1 and MARP2 proteins were strongly expressed both at the RNA level and protein level following UDD (Figure 2E). MARPs have been shown to be important regulators of trophicity and have been proposed as intermediaries between titin and trophic signaling pathways (see Discussion). As the MARPs share high homology and possible redundancy (Miller et al., 2003), we performed 6 day UDD on both single- and multi-KO combinations of the MARPs. We initially performed UDD on the MARP triple KO mice (MARP tKO; knockout of *Ankrd1, –2* and *–23* genes) and found a 12% reduction in the hypertrophy response compared to WT (Figure 4A; *p*=0.0099). This reduction in hypertrophy supports a potential link between titin-mechanosensing and MARP-based trophic signaling. To test if one or a combination of MARPs caused the reduction in hypertrophy, we performed 6 day UDD on MARP1 (*Ankrd1*), MARP2 (*Ankrd2*) and MARP3 (*Ankrd23*) KO mice (Figure 4B–D) and double KO for MARP1/2, MARP1/3 and MARP2/3 (Figure 4—figure supplement 1). Whereas MARP1 KO mice did not show a difference in denervated costal diaphragm mass, MARP2 KO mice showed a 14% increase (*p*=0.003) in mass and MARP3 KO mice showed a 13% reduction (*p*=0.004) in mass gain. However, all MARP double KO mice also presented with attenuated hypertrophy (Figure 4—figure supplement 1). This suggests that the separate MARP proteins could have distinct functions in UDD, and there may be a dependency for two MARPs in regulating trophic signaling, further discussed in the discussion section.

**Figure 4—figure supplement 1.**
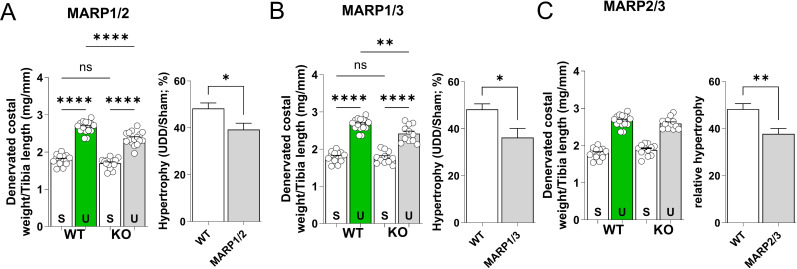
Six-day unilateral diaphragm denervation (UDD) on double knockout (KO) mice of muscle ankyrin repeat proteins (MARPs). Double KO of MARP1/2 (**A**), MARP1/3 (**B**), and MARP2/3 (**C**) all showed a reduction in hypertrophy following UDD, suggesting redundancy between the MARPs. Left panel, diaphragm right costal mass normalized to tibial length and right panel, percentual increase in right costal mass relative to sham. S=Sham, U=UDD (n=10–12). Statistical testing by t-test or two-way ANOVA with Tukey post-hoc testing. Bar graphs denote mean ± SEM.

### MARPs negatively regulate longitudinal hypertrophy

**Figure 5.**
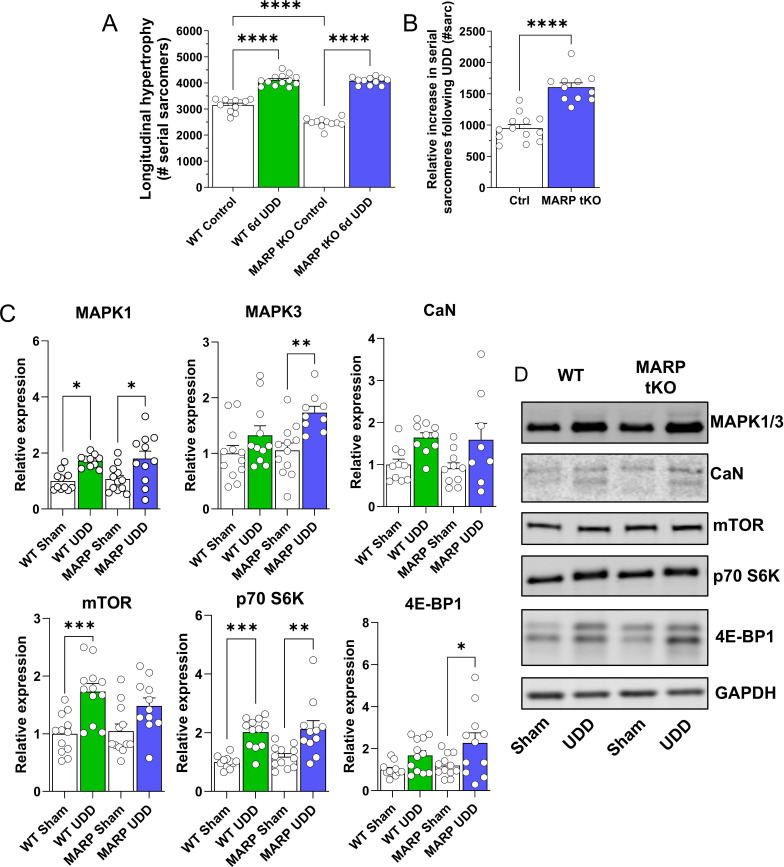
Muscle ankyrin repeat proteins (MARPs) inhibit longitudinal hypertrophy. Longitudinal hypertrophy measured in right costal strips of wild-type (WT) and MARP triple knockout (tKO) mice in 6 day sham and UDD mice (**A**). Numerical increase in serial sarcomeres is higher in MARP tKO (*p*<0.0001) mice compared to WT mice (B; n=11–13), suggesting that the MARPs inhibit longitudinal growth. Probing hypertrophy signaling by western blot, normalized to Gapdh, with expression set relative to WT sham levels (C; n=10–12). Differential mTOR response suggests role in regulating longitudinal hypertrophy. (**D**) Representative blot images of the signaling proteins. Statistical testing by one-way or two-way ANOVA with Tukey post-hoc testing. Bar graphs denote mean ± SEM. Figure 5—source data 1.Unedited western blots. Figure 5—source data 2.Unedited Western blots.

Prompted by the reduction in diaphragm mass gain during UDD in MARP tKO mice and by the complexity of targeting all the single MARP KOs, we assessed longitudinal hypertrophy in 6 day UDD MARP tKO samples. MARP tKO mice at baseline have fewer serial sarcomeres than WT (2476±55 vs 3157±69, respectively; *p*<0.0001), however, 6 days UDD MARP tKO had similar numbers of sarcomeres to WT, 4081±44 vs 4109±59 (Figure 5A). These data show MARP tKO mice add 653±91.6 more sarcomeres than wild-type mice (Figure 5B; 1605±71 vs 952±59, respectively; *p*<0.0001). To discern a possible mechanism of the MARPs inhibiting longitudinal hypertrophy, we probed several candidate proteins of hypertrophy pathways that were prominent in the MS datasets. Western blots for MAPK1/3, Calcineurin, mTOR, P70 S6K and 4E-BP1 were performed on costal diaphragms of WT and MARP tKO, 6 day Sham and UDD animals. All samples were normalized to Gapdh and data were presented as relative to WT sham (Figure 5C; representative western blot images in Figure 5D). Mapk1 showed increases in protein level that were comparable between WT and MARP tKO, whereas MAPK3 showed preferential upregulation in MARP tKO (*p*=0.0099). Calcineurin was unchanged following UDD, note that in a t-test,m the WT mice showed a significant increase in UDD. In WT mice, mTOR showed a striking increase in expression following 6 day UDD (*p*=0.0006), whereas in MARP tKO this was trending (*p*=0.08). Downstream proteins of mTORC1; P70 S6K and 4E-BP1 both showed altered regulation, with P70 S6K being similarly upregulated in WT (*p*=0.0005) versus MARP tKO (*p*=0.001), while 4E-bp1 was only significantly upregulated in MARP tKO (*p*=0.033). Two-way-ANOVA for the signaling proteins did not indicate specific pathway changes between WT and MARP tKO.

### mTOR signaling regulates longitudinal hypertrophy in UDD

**Figure 6.**
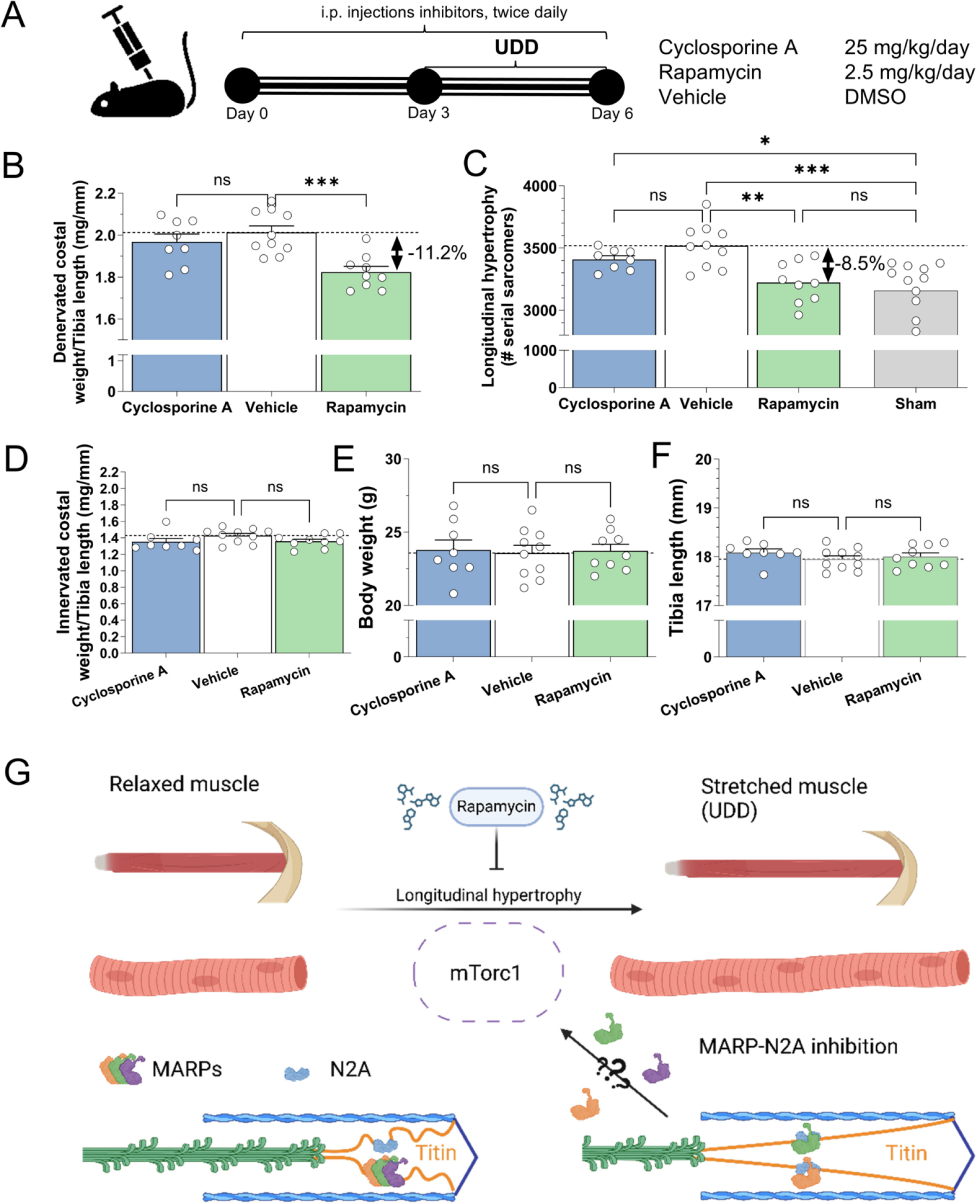
Pharmacological inhibition of the mTOR (rapamycin) and calcium (cyclosporin A) based hypertrophy pathways revealed mTOR to be involved in longitudinal hypertrophy. (**A**) Schematic of the inhibition protocol, showing mice were injected with inhibitors for 3 days prior to receiving unilateral diaphragm denervation (UDD) surgery, with continued twice daily dosing of inhibitors until sacrifice at day 3 post-UDD. Rapamycin inhibited hypertrophy development both at the costal diaphragm mass level (B; *p*<0.001) and at the longitudinal hypertrophy level (C; *p*<0.001), whereas cyclosporin A had no effect. Neither cyclosporin A or rapamycin affected the innervated costal diaphragm (**D**), or body mass (**E**) and all mice used were of approximately the same size based on skeletal size, as measured by tibia length (**F**). (**G**) Hypothetical mechanism for longitudinal hypertrophy following muscle stretch. The mTORC1 pathway is activated by stretch and initiates longitudinal muscle hypertrophy. Muscle ankyrin repeat proteins (MARPs) sequestered by titin’s N2A element are released upon stretch and tune the longitudinal hypertrophy, thus preventing excessive longitudinal hypertrophy. N=8–10/group, statistical testing by one-way-ANOVA and Dunnett’s multiple comparisons test. Bar graphs denote mean ± SEM. Figure 6G was created with BioRender.com.

Motivated by the data from the mTOR western blots in the MARP tKO mice and mTOR being a prominent regulator of skeletal muscle hypertrophy, we performed UDD in WT mice treated with rapamycin, an inhibitor of mTORC1 signaling and skeletal muscle hypertrophy. The rat transcriptome studies also indicated calcium signaling (Supplementary file 1) to be a prominent pathway in UDD. To test the role of calcium signaling, we included a second inhibitor: cyclosporin A, an inhibitor of the calcineurin-NFAT pathway, another hypertrophy-regulating pathway. Mice received twice daily intraperitoneal injections of either rapamycin (2.5 mg/kg/day), cyclosporin A (25 mg/kg/day) or vehicle (DMSO), starting 3 days prior to surgery until sacrifice of the mice (schematic in Figure 6A). Cyclosporin A-treated mice did not show a change in hypertrophy (Figure 6B) or serial sarcomere addition (Figure 6C), indicating that the calcineurin-NFAT pathway is not a primary mechanism for hypertrophy in UDD. Treatment did not affect the innervated left costal diaphragm (Figure 6D), body weight (Figure 6E), or tibia length (Figure 6F). Interestingly, mice that received rapamycin displayed less hypertrophy of the denervated costal diaphragm (Figure 6B; - 11.2%; *p*<0.001) compared to vehicle-treated mice. This attenuated hypertrophy response coincided with a reduction in serial sarcomere addition (Figure 5C; - 8.5%; *p*<0.001) compared to vehicle. Note that following 3 day UDD, rapamycin-treated mice did not show significant increases in serial sarcomeres compared to untreated sham animals (Δ67±81 sarcomeres, versus vehicle-treated mice Δ363±79 sarcomeres). This suggested rapamycin treatment almost completely inhibited longitudinal hypertrophy and that mTORC1 signaling plays a vital role in longitudinal hypertrophy development. We thus propose that mTORC1 signaling positively regulates longitudinal hypertrophy in skeletal muscle and that titin’s N2A-element tunes the extent of longitudinal hypertrophy through the MARPs to prevent excess hypertrophy (graphic summary in Figure 6G).

## Discussion

### Longitudinal hypertrophy as a mechanism for reducing stretch-induced mechanosensing

One of the most striking aspects of the UDD surgical model is the early transient hypertrophy. No other muscle denervation model induces hypertrophy, supporting the notion that stretch, even in inactive muscle, is a potent driver of hypertrophic growth. The extreme nature of the stretch at work in UDD, ~25% stretch of the denervated costal by the innervated costal at a frequency of 120–230 times a minute (respiration rate) (van der Pijl et al., 2018), forms a potent trigger for muscle hypertrophy. Following 6 days UDD, the denervated costal diaphragm develops 49.7±10.0% (Figure 1B) increase in mass, which is primarily caused by addition of 952±81 sarcomeres (Figure 1C) and to a lesser extent by radial fiber growth (van der Pijl et al., 2018). The transient nature of the hypertrophy can be explained by the reduction in sarcomere strain due to the addition of sarcomeres in series (longitudinal hypertrophy), removing the ‘trigger’ that underlies the hypertrophy signaling. This hypothesis fits the data (Figure 1B–C), where before remodeling UDD costal width (i.e. fiber length) is ~8.7 mm (3000 sarcomeres × SL 2.9 µm), and when stretched 25% (van der Pijl et al., 2018) equals a width of ~10.8 mm. Following 6 days of hypertrophic remodeling (UDD), costal width is ~10.8 mm (4000 sarcomeres × SL 2.7 µm). This suggests that the costal width increase attenuates the hypertrophy trigger caused by stretching, finally resulting in atrophy (Figure 1B; 35 day UDD). Denervation itself does not appear to induce hypertrophy of the diaphragm, as 3 day BDD in rat showed no hypertrophy (Figure 1D–E). This does not exclude BDD-mediated denervation from potentially inhibiting or delaying the hypertrophy response. Denervated skeletal muscles have not been reported to experience (longitudinal) hypertrophy. UDD and BDD are both denervation models and hypertrophy occurs specifically in the denervated costal of UDD-operated animals. Stretch is thus the mechanical difference between UDD and BDD and the trigger for hypertrophy signaling. BDD rats do experience abdominal breathing, which could stretch diaphragms sufficiently to induce longitudinal hypertrophy after an extended period of BDD. UDD, being a denervation model, shows that hypertrophy is not necessarily dependent on a muscle’s ability to contract. This indicates that skeletal muscle may derive its signals for hypertrophy during the muscle relaxation phase when the muscle is at its longest state. Muscle stretch in humans is known to benefit muscle growth and is a vital part of exercise routines (reviewed in Nunes et al., 2020; Warneke et al., 2023). Perhaps muscle antagonism may provide sufficient stretch to induce hypertrophy. As contracting muscles inadvertently stretch their relaxed antagonists, they create an elegant feedback loop that promotes both muscle growth and the maintenance of muscle mass.

### Titin’s response to stretch

Titin’s elastic properties have been extensively described (Horowits et al., 1986; Swist et al., 2020). These elastic properties are tied to titin’s role in muscle trophicity, having a direct effect on the extent of muscle hypertrophy (Brynnel et al., 2018; van der Pijl et al., 2018; Strom et al., 2024; Methawasin et al., 2016). We showed that reducing titin stiffness, using both Rbm20 splice-deficient mice and rats, attenuated the stretch-based hypertrophy (Figure 1—figure supplement 3). Importantly, in a model with increased titin stiffness, it was shown that titin stiffness has a direct effect on the number of sarcomeres in series (Brynnel et al., 2018), indicating that titin is a key regulator of longitudinal hypertrophy. Titin splicing analysis by RNA sequencing did not indicate changes in splicing in response to UDD (Figure 1—figure supplement 2). Thus, stretch in rat diaphragm does not alter titin-based stiffness. In contrast, we observed reduced incorporation of exons encoding the PEVK region in BDD rats which likely reflects inactivity promoting increased stiffness of titin. The exact mechanism underlying titin-based regulation of longitudinal hypertrophy is unclear and UDD does not alter titin-stiffness at the splicing level. Thus, we studied phosphorylation as a possible mechanism, focusing on signaling hotspots in titin (reviewed in van der Pijl et al., 2019; van der Pijl et al., 2021a; Voelkel and Linke, 2011) that could mediate longitudinal hypertrophy. Posttranslational changes in titin, particularly phosphorylation, have been studied mostly in relation to passive tension development (Hidalgo and Granzier, 2013; Krüger et al., 2009; Loescher et al., 2022). Specific PTMs for titin have not been widely studied and only recently has ubiquitination been shown to recruit autophagic receptors to the kinase domain of titin (Müller et al., 2021; Bogomolovas et al., 2021). Autophosphorylation of titin kinase at Y170 is considered one of the classic mechanosensing responses in titin resulting in phosphorylation of the autophagic receptor Nbr1 at S115/116, activating autophagy signaling in vitro (Lange et al., 2005). We did not observe any signs following UDD that titin kinase was autophosphorylated at Y170. This could be related to phospho-peptide abundance being below the detection limit, or that the titin kinase is inactive (Bogomolovas et al., 2014). In total, we identified ~700 phosphorylation sites in titin of which 142 were significantly affected by stretch. These sites were distributed along the entire length of titin with several hot spots in the PEVK region, likely involved in stiffness regulation, and several in the Z-disk, which could be related to signaling or structural interactions. The sites in the N2A-element (Figure 3) were of particular interest, as previously it has been suggested that S9540 (S9895 according to diaphragm RNA-seq by Brynnel et al., 2018) could serve as a recruitment signal for MARP1 (Lanzicher et al., 2020), a protein that is highly upregulated following UDD (Figure 2E). MARPs have been shown to quench N2A phosphorylation (Lanzicher et al., 2020; Lun et al., 2014), which make the phosphorylation sites in the N2A segment tantalizing targets for studying titin-MARP binding. If S9459 and S9520 (Figure 3D–E) play such a role remains to be determined, but such insights could provide future avenues for manipulating titin-MARP interactions. Similarly, we found many titin-associated proteins to show differential phosphorylation in UDD, including three sites in the MARPs (Figure 3—figure supplement 1). How these sites contribute to signaling or recruitment to the N2A-element remains to be seen but provide tantalizing targets for follow-up study.

### The MARP proteins and muscle trophicity

Global transcriptome and proteome studies from 3 day UDD showed multiple titin-associated proteins being upregulated (Figure 2E). We focused our efforts on the MARP proteins, as they are known interacting partners of titin’s N2A-element and have been shown to be important for hypertrophy regulation in the heart (Zhong et al., 2015; Lange et al., 2016). We used UDD on MARP KO mice, focusing on the triple KO for MARP1-3 to account for expected redundancy (Miller et al., 2003; Lun et al., 2014). MARP tKO showed a 12% reduction in hypertrophy following 6 day UDD (Figure 4A). This suggested stretch-mechanosensing operated through the MARP proteins. In an attempt to isolate a single MARP protein as the main effector, we performed UDD on single MARP knock-out mice (Figure 4B–D). MARP1 KO did not reveal changes in hypertrophy. However, we previously established that MARP1 localizes to the N2A-segment of titin following UDD (van der Pijl et al., 2018). We also independently determined that MARP1 cross-links titin to the thin filament to increase passive tension (van der Pijl et al., 2021b; Zhou et al., 2021), suggesting MARP1 plays mechanical roles over trophic regulation in skeletal muscle. MARP2 KO developed an exaggerated hypertrophy and lastly, MARP3 KO showed an attenuated hypertrophy response to UDD, suggesting MARP2 and 3 play opposing roles in hypertrophy regulation. MARP2 interacts with Akt2 (Cenni et al., 2011), providing a tentative link with the mTOR pathway, discussed below. Additionally, MARP2 interaction with P50-NFκB acting as an analogue for IκB (Bean et al., 2014) suggests a possible role in inhibiting NFκB atrophy signaling. The role of MARP3 remains incompletely understood, but prior studies indicate that loss of MARP3 enhances glucose tolerance and insulin sensitivity (Shimoda et al., 2015), and MARP3 has been linked to AMPK signaling. AMPK is a key regulator of metabolic pathways and a well-established inhibitor of hypertrophic signaling, in part through suppression of mTOR activity, and is also responsive to mechanical stimuli (Budinger et al., 2008; Shimoda et al., 2015). To specifically determine if the MARPs affected longitudinal hypertrophy we measured the number of serial sarcomeres across the width of the costal diaphragm in MARP tKO and found that the tKO mice added 653.2±91.55 more sarcomeres than WT following 6 day UDD (Figure 5B). This suggests that the MARP proteins inhibit longitudinal hypertrophy. A possible mechanism could be that following stretch, the three MARPs bind and compete for the N2A-binding site (Miller et al., 2003; Lun et al., 2014) resulting in translocation of specific MARPs for signaling purposes. The benefit of MARP deletion in cardiomyopathy was shown by Lange et al., 2016, where deletion of MARP1/2 ameliorated MLP KO-induced DCM. MARP KO has not proven detrimental in mice (Barash et al., 2007), making this family an attractive target for therapeutic intervention.

### mTOR and longitudinal hypertrophy

Muscle hypertrophy is regulated through a number of pathways, with the insulin-insulin growth factor (IGF) mediated pathway (Schiaffino and Mammucari, 2011; Goodman, 2019; Yoon, 2017) being the most well understood in skeletal muscle and the calcineurin-NFAT pathway in cardiac muscle (Molkentin, 2004; Molkentin, 2013). With most studies focused on radial (cross-sectional) hypertrophy, we aimed to gain insight into the regulatory mechanism underlying longitudinal hypertrophy, two types of hypertrophy that are not necessarily mutually exclusive. The mTOR signaling pathway was a prime candidate as UDD in MARP tKO mice suggested altered mTOR activity (Figure 5C–D). This prompted us to test inhibition of mTOR signaling and see if mTOR regulates longitudinal hypertrophy. Using rapamycin (Li et al., 2014), an inhibitor that targets the mTORC1 (protein synthesis regulation) complex, we found a reduction in longitudinal hypertrophy following 3 days UDD compared to vehicle-treated mice (Figure 6B). This strongly suggests that the mTORC1 pathway is in part responsible for longitudinal hypertrophy following UDD. While rapamycin primarily inhibits mTORC1, rapamycin-insensitive signaling through mTORC2 may also contribute to UDD stretch-induced responses. Denervation itself may activate mTORC2-associated signaling pathways, which could confound the interpretation of stretch-specific versus denervation-mediated mTOR responses in this model. Therefore, a contribution of rapamycin-insensitive mTOR signaling to the observed phenotype cannot be excluded. Although we focused on mTOR signaling in longitudinal hypertrophy development, we do not exclude other pathways being important. mTOR formed an attractive target as previous work showed that longitudinal stretch phosphorylates Akt and upregulates MARP2 (Mohamed et al., 2010), forming a tentative link between longitudinal stretch, MARPs and the mTOR pathway. mTORC1-based hypertrophy is primarily mediated through P70 S6K and 4E-BP1 and their respective phosphorylation signals at T389 and T37/46 (Goodman, 2019; Yoon, 2017). These sites were unchanged at 24 hr UDD (Supplementary file 3) indicating that these sites are activated at a later stage, as reported by Norrby et al., 2012, or that mTOR is activated through alternative pathways following stretch. Further studies are needed to establish the roles of the various pathways in stretch hypertrophy.

In conclusion, we found that the transient hypertrophy induced by UDD is dependent on muscle fiber length, with longitudinal hypertrophy reducing the trigger for hypertrophy. The hypertrophy coincides with increased phosphorylation of the N2A element. The N2A-associated MARP proteins are strongly upregulated following UDD and deletion of the MARPs increases the extent of longitudinal hypertrophy following UDD, indicating the MARPs serve roles in inhibiting longitudinal hypertrophy. MARP tKO mice show altered regulation in the mTORC1 pathway following UDD and inhibition of mTORC1 by rapamycin shows mTOR is a main regulator of longitudinal hypertrophy.

## Methods

**Key resources table:** 

Reagent type (species) or resource	Designation	Source or reference	Identifiers	Additional information
Gene (*Mus musculus*)	*Ttn*	NCBI Gene	Gene ID: 22138	Titin
Gene (*Mus musculus*)	*Ankrd1*	NCBI Gene	Gene ID: 107765	MARP1
Gene (*Mus musculus*)	*Ankrd2*	NCBI Gene	Gene ID: 107766	MARP2
Gene (*Mus musculus*)	*Ankrd23*	NCBI Gene	Gene ID: 64009	MARP3 /DARP
Genetic reagent (*Mus musculus*)	MARP1 KO (*Ankrd1* KO)	Bang et al., 2014	https://doi.org/10.1371/journal.pone.0093638	Used for UDD studies
Genetic reagent (*Mus musculus*)	MARP2 KO (*Ankrd2* KO)	Bang et al., 2014	https://doi.org/10.1371/journal.pone.0093638	Used for UDD studies
Genetic reagent (*Mus musculus*)	MARP3 KO (*Ankrd23* KO)	Bang et al., 2014	https://doi.org/10.1371/journal.pone.0093638	Used for UDD studies
Genetic reagent (*Mus musculus*)	MARP Triple KO	Barash et al., 2007	https://doi.org/10.1152/ajpcell.00055.2007	Used for UDD studies
Genetic reagent (*Mus musculus*)	Rbm20^ΔRRM^	Methawasin et al., 2014	https://doi.org/10.1161/CIRCULATIONAHA.113.005610	Used for UDD studies
Genetic reagent (*Rattus norvegicus*)	Sprague Dawley	Charles River	Crl:CD(SD)	Used for BDD/UDD studies
Genetic reagent (*Rattus norvegicus*)	Rbm20 KO	Li et al., 2013	https://doi.org/10.1093/nar/gks1362	Used for UDD studies
Commercial assay, kit	RNeasy Fibrous Tissue Mini Kit	Qiagen	Cat#74704	RNA isolation
Commercial assay, kit	TruSeq RNA Library Prep Kit v2	Illumina	RS-122–2001	RNA-seq library preparation
Commercial assay, kit	Total RNA Prep, Ligation with Ribo-Zero Plus	Illumina	20040525	rRNA depletion before library prep
Commercial assay, kit	Pierce Quantitative Colorimetric Peptide Assay Kit	Thermo Fisher	Cat#23275	Peptide quantification
Commercial assay, kit	High-Select Fe-NTA Phosphopeptide Enrichment Kit	Thermo Fisher	Cat#A32992	Phosphopeptide enrichment
Commercial assay, kit	High-Select TiO2 Phosphopeptide Enrichment Kit	Thermo Fisher	Cat#A32993	Phosphopeptide enrichment
Antibody	anti-MAPK1/3 (Mouse IgG1)	Cell Signaling	Cat#4696	WB (1:200)
Antibody	anti-Calcineurin (Mouse IgG2a)	BD Biosciences	Cat#610260	WB (1:750)
Antibody	anti-mTOR (Rabbit IgG)	Cell Signaling	Cat#2983	WB (1:750)
Antibody	anti-P70S6K (Rabbit IgG)	Cell Signaling	Cat#2708	WB (1:500)
Antibody	anti-4EBP1 (Rabbit IgG)	Cell Signaling	Cat#9452	WB (1:750)
Antibody	anti-GAPDH (Rabbit IgG)	Cell Signaling	Cat#2112	WB (1:5000)
Antibody	anti-GAPDH (Mouse IgG1)	Thermo Fisher	Cat#MA5-15738	WB (1:3000)
Chemical compound, drug	Rapamycin	Selleck Chem	S1039	2.5 mg/kg/day
Chemical compound, drug	Cyclosporin A	Selleck Chem	S2286	25 mg/kg/day
Software, algorithm	STAR	Dobin et al.	RRID:SCR_015899	RNA-seq alignment
Software, algorithm	DESeq2	Love et al.	RRID:SCR_015687	Differential expression
Software, algorithm	Progenesis QI for Proteomics	Nonlinear Dynamics	RRID:SCR_018923	Proteomics analysis, Version 2.4
Software, algorithm	Perseus	Tyanova et al., 2016	RRID:SCR_015753	Proteomics analysis, Version 2.0.3.1
Software, algorithm	GraphPad Prism	GraphPad	RRID:SCR_002798	Statistics, Version 9.1
Software, algorithm	ShinyGO	Ge et al., 2020	RRID:SCR_019213	GO analysis, Version 0.75
Software, algorithm	Heatmapper	Babicki et al., 2016	RRID:SCR_016974	Heatmap generation
Software, algorithm	BioVenn	Hulsen et al., 2008	RRID:SCR_026853	Venn analysis

### Animal studies

All experiments were done in accordance with the University of Arizona Institutional Animal Care and Use Committee and followed the US National Institutes of Health Using Animals in Intramural Research guidelines for animal use (protocol 13–488). We used 3-month-old C57BL/6 J mice referred to as wild-type (WT), and 6-month-old Sprague-Dawley rats (SD). Knockout models for titin binding proteins MARP1-3 (*Ankrd1*, *Ankrd2*, and *Ankrd23* KO) (Lange et al., 2016; Barash et al., 2007; Bang et al., 2014) were kindly provided by Dr Ju Chen and Dr Stephan Lange. Homozygous Rbm20^ΔRRM^ mice (van der Pijl et al., 2018; Methawasin et al., 2014) and Rbm20 KO rats (Li et al., 2013; Lindqvist et al., 2018), have previously been described. Mice were maintained on a C57BL/6 J background, with the data from the MARP mice being on a black Swiss background.

#### Surgical procedure

For unilateral diaphragm surgery (UDD) (van der Pijl et al., 2018) or bilateral diaphragm denervation (BDD) studies, mice or rats were anaesthetized with 2–3% isoflurane and a small incision was made in the neck area just above the clavicle. The right phrenic nerve was isolated behind the sternohyoid muscle, and a 3–4 mm section was transected at the height of the supraclavicular nerve branch. For BDD surgery both left and right phrenic nerves being transected. Sham-operated animals underwent the same procedure, except the phrenic nerve was left intact. Animals were sacrificed 1, 3, 6, 12, or 35 days after surgery for morphometric analysis and tissue harvest.

### Pharmacological inhibition of hypertrophy

Inhibitor studies with rapamycin (mTOR inhibitor) and cyclosporin A (Calcineurin inhibitor) were performed by injecting mice, twice daily intraperitoneal (IP), with 2.5 or 25 mg/kg/day, respectively. Each inhibitor was dissolved in dimethyl sulfoxide (DMSO) and diluted to 20% DMSO with saline solution just before injection. Mice received inhibitors or vehicle (20% DMSO in saline) starting 3 days prior to UDD surgery to prime the mice and continued following UDD to inhibit hypertrophy growth (Figure 4C). Mice were sacrificed after 3 days ofUDD and processed for serial sarcomere measurements as described below.

### Serial sarcomere measurements

Previously described in van der Pijl et al., 2018. Briefly, mice were anaesthetized with a 140/10 mg/kg ketamine/xylazine solution, a small incision to visualize the jugular vein, which was subsequently cannulated for perfusion. Mice were perfused with a solution consisting of 4% formaldehyde, with 70 U/ml of heparin in phosphate-buffered saline (PBS), after which the diaphragm was removed and stored in 4% formaldehyde in PBS overnight for complete fixation. Full-length diaphragm midcostal strips were gently dissected and flattened between glass slides, costal width was measured using a caliper and sarcomere lengths were measured using a He/Ne laser diffraction system.

Alternatively, muscle fiber bundles from chemically demembranated full-length diaphragm midcostal strips of 3 day mice were flattened between glass slides. Costal width and sarcomere lengths were measured using a Zeiss Axio Imager M1 microscope (Zeiss), at 50× magnification to measure the length of the muscle bundles and 640× for sarcomere length along four points of the fiber bundles. Images were captured using AxioCam MRc with Axiovision software (Zeiss) and images were calibrated using a 0.01 mm stage micrometre (Edmund Optics). To determine the number of serial sarcomeres, the muscle bundle length (costal width) was divided by the sarcomere length.

Demembranating solution consisted of a relaxing solution (in mM; 20 BES, 10 EGTA, 6.56 MgCl2, 5.88 NaATP, 1 DTT, 46.35 K‐propionate, 15 creatine phosphate, pH 7.0), with 1% Triton‐X‐100 at 4 °C, and protease inhibitors (phenylmethylsulfonyl fluoride (PMSF), 0.25 mM; leupeptin, 0.04 mM; E64, 0.01 mM), after demembranation, samples were stored in just relaxing solution plus inhibitors (without triton X-100) at 4 °C.

### Transcriptome studies

RNA sequencing (RNA-seq) was performed on right costal diaphragm samples collected from 3 day sham and UDD animals and flash frozen in liquid nitrogen until further processing. For RNA extraction, samples were incubated overnight in RNAlater-ICE (Thermo Scientific) and subsequently transferred to RLT buffer for extraction according to the RNeasy Fibrous Tissue Mini Kit (Qiagen). Tissue disruption was achieved using a Bullet Blender (Next Advance) and Green Eppendorf lysis kit tubes (Next Advance), by grinding samples for 4 min at setting 10. Thereafter, total RNA extraction was performed following the RNeasy Fibrous Tissue Mini Kit’s instructions and quantified using a Nanodrop ND-1000 spectrophotometer (Thermo Scientific). Each sample consisted of three biological replicate sham or UDD samples. Both library preparation and sequencing were performed by the Novogene, Sacramento, USA. Briefly, library preparation: rRNA was depleted from RNA preparations from 1 µg total RNA. Libraries were prepared using an RNA Library Prep Kit from Illumina following the manufacturer’s instructions. Sequencing was performed on an Illumina NovaSeq X sequencer using 150 bp paired-end sequencing. For RNA-seq analysis see Brynnel et al., 2018. Briefly, adapters and low-quality reads were removed with Trim Galore and reads were mapped to the rat genome (Release mRatBN7.2) using STAR (Dobin et al., 2013) with default settings. Differentially expressed genes were determined with DESeq2 (Love et al., 2014). Genes with population adjusted *p*-values (p_adj_)<0.05 were considered differentially expressed. For titin splicing, percent spliced in index (PSI) was calculated as a measure for determining if an exon is spliced in, following the titin exon annotation by Bang et al., 2001. RNA sequence data were deposited at NIH NCBI BioProject (accession number PRJNA1460879).

### Preparation of muscle for mass spectrometry analysis

Diaphragm muscle was ground to a fine powder using Dounce homogenizers cooled in liquid nitrogen and acclimated to –20 °C for 30 min before continuing. Tissue powder was resuspended at a concentration of 50 mg/ml in a urea buffer (4 M urea, 1 M thiourea, 25 mM Tris–HCl, 75 mM dithiothreitol, 1.5% SDS, 25% glycerol, pH 6.8) with protease inhibitors (0.04 mM E‐64, 0.16 mM leupeptin, and 0.2 mM PMSF). The solution was mixed for 4 min, followed by 10 min of incubation at 60 °C. Samples were centrifuged at 12,000 rpm and the supernatant flash frozen for storage at −80 °C.

### In-solution tryptic digestion

50 µg of rat costal diaphragm lysate was subjected to acetone precipitation by adding six times the sample volume of pre-chilled 100% acetone and incubated 1 hr at –20 °C. The precipitates were centrifuged at 16,000 × g for 10 min at 4 °C and the acetone was removed. 400 µL of pre-chilled 90% acetone was added to the protein pellet, briefly vortexed and centrifuged at 16,000 × g for 5 min at 4 °C. The remaining acetone was removed, the protein pellets were air dried for 3 min, resuspended in 100 µl of 50 mM NH_4_HCO_3_, and sonicated for 5 min. The samples were supplemented with dithiothreitol (DTT) at a final concentration of 5 mM and incubated at 70 °C for 30 min. Samples were cooled to room temperature for 10 min and incubated with 15 mM acrylamide for 30 min at room temperature while protected from light. The reaction was quenched with DTT with a final concentration of 5 mM and incubated in the dark for 15 min. One µg of Lys-C was added to each sample and incubated at 37 ° C for 2–3 hr while shaking at 300 rpm followed by the addition of 50 µl of 50 mM ammonium bicarbonate and 2 µg of trypsin and incubation overnight at 37 °C while shaking at 300 rpm. 14.7 µl of 40% FA/1% HFBA was added to each sample and incubated for 10 min (final concentration is 4% FA/0.1% HFBA) to stop trypsin digestion. The samples were desalted with Pierce Peptide Desalting Spin Columns per the manufacturer’s protocol (Thermo Fisher Scientific, cat no. 89852) and the peptides were dried by vacuum centrifugation. The dried peptides were resuspended in 20 µl of 0.1% FA (vol/vol) and the peptide concentration was determined with the Pierce Quantitative Colorimetric Peptide Assay Kit per the manufacturer’s protocol (Thermo Fisher Scientific, cat no. 23275). 350 ng of the final sample was analyzed by mass spectrometry.

### Phosphoproteomics

To determine global differences in protein phosphorylation abundance between sham or UDD, 1 mL of protein lysate corresponding to 50 mg diaphragm per sample (pooled costal diaphragm of 2 mice) was subjected to in-solution tryptic digestion and phosphopeptide enrichment using sequential enrichment from metal oxide affinity chromatography per manufacturer’s protocol (Thermo Scientific, cat no. A32993 & A32992) similar to as previously described (Levine et al., 2023; Keresztes et al., 2022). The dried peptides were resuspended in 20 µl of 0.1% FA (vol/vol) and the peptide concentration was determined with the Pierce Quantitative Colorimetric Peptide Assay Kit per the manufacturer’s protocol. 350 ng of the final sample was then analyzed by mass spectrometry.

### Mass spectrometry

HPLC-ESI-MS/MS was performed in positive ion mode on a Thermo Scientific Orbitrap Fusion Lumos tribrid mass spectrometer fitted with an EASY-Spray Source (Thermo Scientific, San Jose, CA). NanoLC was performed using a Thermo Scientific UltiMate 3000 RSLCnano System with an EASY Spray C18 LC column (Thermo Scientific, 50 cm×75 μm inner diameter, packed with PepMap RSLC C18 material, 2 µm, cat. # ES803); loading phase for 15 min at 0.300 µl/min; mobile phase, linear gradient of 1–34% Buffer B in 119 min at 0.220 µl/min, followed by a step to 95% Buffer B over 4 min at 0.220 µl/min, hold 5 min at 0.250 µl/min, and then a step to 1% Buffer B over 5 min at 0.250 µl/min and a final hold for 10 min (total run 159 min); Buffer A=0.1% FA/H_2_O; Buffer B=0.1% FA in 80% ACN. All solvents were liquid chromatography mass spectrometry grade. Spectra were acquired using XCalibur, version 2.3 (Thermo Fisher Scientific). A ‘TopSpeed’ data-dependent MS/MS analysis was performed (acquisition of a full scan spectrum followed by collision-induced dissociation mass spectra of the Top N most intense precursor ions within the 3 s cycle time). Dynamic exclusion was enabled with a repeat count of 1, a repeat duration of 30 s, an exclusion list size of 500, and an exclusion duration of 40 s.

### Label-free quantitative proteomics

Progenesis QI for proteomics software (version 2.4, Nonlinear Dynamics Ltd., Newcastle upon Tyne, UK) was used to perform ion-intensity-based label-free quantification as previously described (Parker et al., 2019). In brief, in an automated format, raw files were imported and converted into two-dimensional maps (y-axis=time, x-axis=m/z) followed by selection of a reference run for alignment purposes. An aggregate data set containing all peak information from all samples was created from the aligned runs, which was then further narrowed down by selecting only +2, +3, and +4 charged ions for further analysis. The samples were then grouped according to treatment. Peak lists of the top ten fragment ion spectra were exported in Mascot generic file (mgf) format and searched against either the 2020_06 Swiss-Prot *Rattus norvegicus* database (8128 entries), the 2018_11 Swiss-Prot *Mus musculus* database (17008 entries), or species respective TrEMBL databases using Mascot (Matrix Science, London, UK; version 2.6.0). The search variables that were used were: 10 ppm mass tolerance for precursor ion masses and 0.5 Da for product ion masses; digestion with trypsin; a maximum of two missed tryptic cleavages; variable modifications of oxidation of methionine, phosphorylation of serine, threonine, and tyrosine, and carbamidomethylation of cysteine; 13C=1. The resulting Mascot.xml file was then imported into Progenesis, allowing for peptide/protein assignment, while peptides with a Mascot Ion Score of <25 were not considered for further analysis. False discovery rate was set to 1% for both peptides (minimum length of seven amino acids) and proteins. Abundances were normalized to the total ion current (TIC) to correct for differences in sample loading and instrument response. For quantification, proteins must have possessed at least one or more unique, identifying peptide.

### Visualization and analysis of transcriptome and proteome data

Figures were generated using online resources, briefly, quantitative Venn diagrams were made using https://www.biovenn.nl/ (Hulsen et al., 2008). Lolipop graphs of gene ontology (GO) enrichment analysis by ShinyGO v0.75 http://bioinformatics.sdstate.edu/go/ (Ge et al., 2020). Heatmaps were generated using a combination of Graphpad Prism v9.1, Perseus v2.0.3.1 (Tyanova et al., 2016) and Heatmapper (http://heatmapper.ca/) (Babicki et al., 2016). Perseus was also used to analyze principal components and visualized using Graphpad Prism. A protein mapping table was constructed in R, using the R Interface to UniProt Web Services package version 2.34.0 (Carlson et al., 2021). UniProtKB identifiers and gene IDs were assigned to each peptide. Phosphorylated peptides were mapped to the UniProt sequences to identify the positions of the modified residue(s) using stringr version 1.4.0 (Wickham, 2019). For representation of titin phosphorylation, normalized abundance values were converted to Z-scores by subtracting the grand-mean of normalized abundance values for a particular peptide and dividing by the grand-mean standard deviation. We subsequently summed Z-scores of isobaric peptides for a given phospho-site to generate values to represent expression levels. To generate domain or regional phosphorylation, we further summed phospho-sites to derive domain and regional phosphorylation graphs.

### Western blot

Western blot experiments were previously described in van der Pijl et al., 2018. Proteins were transferred onto Immobilon-P PVDF 0.45 μm membranes (Millipore) using semi-dry transfer (Bio-Rad). Membranes were blocked with Odyssey blocking buffer (Li-Cor Biosciences) for 1 hr, and subsequently probed with primary antibodies at 4 °C overnight (See Table 1). Near Infra-Red dyes were used as secondary antibodies for detection with Odyssey CLx Imaging System (Li-Cor Biosciences, United States).

**Table 1.** Antibodies used in this study.

Antibody	Source/isotype	Dilution	Company	Catalog#
MAPK1/3 (ERK2/1)	Mouse IgG1	1:200	Cell Signaling	#4696
Calcineurin	Mouse IgG2a	1:750	BD Biosciences	610260
mTOR	Rabbit IgG	1:750	Cell Signaling	#2983
P70S6K	Rabbit IgG	1:500	Cell Signaling	#2708
4EBP1	Rabbit IgG	1:750	Cell Signaling	#9452
Gapdh	Rabbit IgG	1:5000	Cell Signaling	#2112
Gapdh	Mouse IgG1	1:3000	Thermo Fisher Scientific.	MA5-15738
CF790 Goat anti Mouse IgG	Goat IgG	1:10,000	Biotium	20342
CF680 Goat anti Mouse IgG	Goat IgG	1:10,000	Biotium	20065
CF680 Goat anti Rabbit IgG	Goat IgG	1:10,000	Biotium	20067
CF680R Goat anti Mouse IgG2a	Goat IgG	1:10,000	Biotium	20842

## Data Availability

Sequence data were deposited in BioProject (accession number PRJNA1460879). All data generated or analyzed during this study are included in the manuscript and supporting files. The following dataset was generated: van der PijlR GohlkeJ StromJ PetersE ShenS ConijnS HouraniZ LangeS ChenJ LanglaisP LabeitS GranzierH OttenheijmC 2026The titin N2A-MARP signalosome constrains muscle longitudinal hypertrophy in response to stretchNCBI BioProjectPRJNA146087910.7554/eLife.107597PMC1342335542530086

## Additional files

MDAR checklist

Supplementary file 1. Rat RNAseq analysis.

Supplementary file 2. Rat Global mass spectrometry analysis.

Supplementary file 3. Mouse Phosphorylation mass spectrometry analysis.

## References

[bib1] Babicki S, Arndt D, Marcu A, Liang Y, Grant JR, Maciejewski A, Wishart DS (2016). Heatmapper: web-enabled heat mapping for all. Nucleic Acids Research.

[bib2] Bang ML, Centner T, Fornoff F, Geach AJ, Gotthardt M, McNabb M, Witt CC, Labeit D, Gregorio CC, Granzier H, Labeit S (2001). The complete gene sequence of titin, expression of an unusual approximately 700-kDa titin isoform, and its interaction with obscurin identify a novel Z-line to I-band linking system. Circulation Research.

[bib3] Bang ML, Gu Y, Dalton ND, Peterson KL, Chien KR, Chen J (2014). The muscle ankyrin repeat proteins CARP, Ankrd2, and DARP are not essential for normal cardiac development and function at basal conditions and in response to pressure overload. PLOS ONE.

[bib4] Barash IA, Bang ML, Mathew L, Greaser ML, Chen J, Lieber RL (2007). Structural and regulatory roles of muscle ankyrin repeat protein family in skeletal muscle. American Journal of Physiology. Cell Physiology.

[bib5] Bean C, Verma NK, Yamamoto DL, Chemello F, Cenni V, Filomena MC, Chen J, Bang ML, Lanfranchi G (2014). Ankrd2 is a modulator of NF-κB-mediated inflammatory responses during muscle differentiation. Cell Death & Disease.

[bib6] Bertz M, Wilmanns M, Rief M (2009). The titin-telethonin complex is a directed, superstable molecular bond in the muscle Z-disk. PNAS.

[bib7] Bogomolovas J, Gasch A, Simkovic F, Rigden DJ, Labeit S, Mayans O (2014). Titin kinase is an inactive pseudokinase scaffold that supports MuRF1 recruitment to the sarcomeric M-line. Open Biology.

[bib8] Bogomolovas J, Fleming JR, Franke B, Manso B, Simon B, Gasch A, Markovic M, Brunner T, Knöll R, Chen J, Labeit S, Scheffner M, Peter C, Mayans O (2021). Titin kinase ubiquitination aligns autophagy receptors with mechanical signals in the sarcomere. The EMBO Reports.

[bib9] Brynnel A, Hernandez Y, Kiss B, Lindqvist J, Adler M, Kolb J, van der Pijl R, Gohlke J, Strom J, Smith J, Ottenheijm C, Granzier HL (2018). Downsizing the molecular spring of the giant protein titin reveals that skeletal muscle titin determines passive stiffness and drives longitudinal hypertrophy. eLife.

[bib10] Budinger GRS, Urich D, DeBiase PJ, Chiarella SE, Burgess ZO, Baker CM, Soberanes S, Mutlu GM, Jones JCR (2008). Stretch-induced activation of AMP kinase in the lung requires dystroglycan. American Journal of Respiratory Cell and Molecular Biology.

[bib11] Carlson M, Ortutay C, Ramos M (2021). Interface to uniprot web services. Bioconductor.

[bib12] Cenni V, Bavelloni A, Beretti F, Tagliavini F, Manzoli L, Lattanzi G, Maraldi NM, Cocco L, Marmiroli S (2011). Ankrd2/ARPP is a novel Akt2 specific substrate and regulates myogenic differentiation upon cellular exposure to H(2)O(2). Molecular Biology of the Cell.

[bib13] Dobin A, Davis CA, Schlesinger F, Drenkow J, Zaleski C, Jha S, Batut P, Chaisson M, Gingeras TR (2013). STAR: ultrafast universal RNA-seq aligner. Bioinformatics.

[bib14] Ge SX, Jung D, Yao R (2020). ShinyGO: a graphical gene-set enrichment tool for animals and plants. Bioinformatics.

[bib15] Goodman CA (2019). Role of mTORC1 in mechanically induced increases in translation and skeletal muscle mass. Journal of Applied Physiology.

[bib16] Hayashi C, Ono Y, Doi N, Kitamura F, Tagami M, Mineki R, Arai T, Taguchi H, Yanagida M, Hirner S, Labeit D, Labeit S, Sorimachi H (2008). Multiple molecular interactions implicate the connectin/titin N2A region as a modulating scaffold for p94/calpain 3 activity in skeletal muscle. The Journal of Biological Chemistry.

[bib17] Hidalgo C, Granzier H (2013). Tuning the molecular giant titin through phosphorylation: role in health and disease. Trends in Cardiovascular Medicine.

[bib18] Horowits R, Kempner ES, Bisher ME, Podolsky RJ (1986). A physiological role for titin and nebulin in skeletal muscle. Nature.

[bib19] Hulsen T, de Vlieg J, Alkema W (2008). BioVenn - a web application for the comparison and visualization of biological lists using area-proportional Venn diagrams. BMC Genomics.

[bib20] Keresztes A, Olson K, Nguyen P, Lopez-Pier MA, Hecksel R, Barker NK, Liu Z, Hruby V, Konhilas J, Langlais PR, Streicher JM (2022). Antagonism of the mu-delta opioid receptor heterodimer enhances opioid antinociception by activating Src and calcium/calmodulin-dependent protein kinase II signaling. Pain.

[bib21] Krüger M, Kötter S, Grützner A, Lang P, Andresen C, Redfield MM, Butt E, dos Remedios CG, Linke WA (2009). Protein kinase G modulates human myocardial passive stiffness by phosphorylation of the titin springs. Circulation Research.

[bib22] Labeit S, Kolmerer B (1995). Titins: giant proteins in charge of muscle ultrastructure and elasticity. Science.

[bib23] Lange S, Xiang F, Yakovenko A, Vihola A, Hackman P, Rostkova E, Kristensen J, Brandmeier B, Franzen G, Hedberg B, Gunnarsson LG, Hughes SM, Marchand S, Sejersen T, Richard I, Edström L, Ehler E, Udd B, Gautel M (2005). The kinase domain of titin controls muscle gene expression and protein turnover. Science.

[bib24] Lange S, Gehmlich K, Lun AS, Blondelle J, Hooper C, Dalton ND, Alvarez EA, Zhang X, Bang M-L, Abassi YA, Dos Remedios CG, Peterson KL, Chen J, Ehler E (2016). MLP and CARP are linked to chronic PKCα signalling in dilated cardiomyopathy. Nature Communications.

[bib25] Lanzicher T, Zhou T, Saripalli C, Keschrumrus V, Smith Iii JE, Mayans O, Sbaizero O, Granzier H (2020). Single-molecule force spectroscopy on the N2A element of titin: effects of phosphorylation and CARP. Frontiers in Physiology.

[bib26] Laughlin MH, Yang HT, Tharp DL, Rector RS, Padilla J, Bowles DK (2017). Vascular cell transcriptomic changes to exercise training differ directionally along and between skeletal muscle arteriolar trees. Microcirculation.

[bib27] Levine AA, Liktor-Busa E, Balasubramanian S, Palomino SM, Burtman AM, Couture SA, Lipinski AA, Langlais PR, Largent-Milnes TM (2023). Depletion of endothelial-derived 2-AG reduces blood-endothelial barrier integrity via alteration of VE-cadherin and the phospho-proteome. International Journal of Molecular Sciences.

[bib28] Li S, Guo W, Dewey CN, Greaser ML (2013). Rbm20 regulates titin alternative splicing as a splicing repressor. Nucleic Acids Research.

[bib29] Li J, Kim SG, Blenis J (2014). Rapamycin: one drug, many effects. Cell Metabolism.

[bib30] Lindqvist J, van den Berg M, van der Pijl R, Hooijman PE, Beishuizen A, Elshof J, de Waard M, Girbes A, Spoelstra-de Man A, Shi ZH, van den Brom C, Bogaards S, Shen S, Strom J, Granzier H, Kole J, Musters RJP, Paul MA, Heunks LMA, Ottenheijm CAC (2018). Positive end-expiratory pressure ventilation induces longitudinal atrophy in diaphragm fibers. American Journal of Respiratory and Critical Care Medicine.

[bib31] Loescher CM, Hobbach AJ, Linke WA (2022). Titin (TTN): from molecule to modifications, mechanics, and medical significance. Cardiovascular Research.

[bib32] Love MI, Huber W, Anders S (2014). Moderated estimation of fold change and dispersion for RNA-seq data with DESeq2. Genome Biology.

[bib33] Lun AS, Chen J, Lange S (2014). Probing muscle ankyrin‐repeat protein (MARP) structure and function. The Anatomical Record.

[bib34] Mayans O, van der Ven PF, Wilm M, Mues A, Young P, Fürst DO, Wilmanns M, Gautel M (1998). Structural basis for activation of the titin kinase domain during myofibrillogenesis. Nature.

[bib35] Methawasin M, Hutchinson KR, Lee EJ, Saripalli C, Hidalgo CG, Ottenheijm CAC, Granzier H (2014). Experimentally increasing titin compliance in a novel mouse model attenuates the Frank-Starling mechanism but has a beneficial effect on diastole. Circulation.

[bib36] Methawasin M, Strom JG, Slater RE, Fernandez V, Saripalli C, Granzier HL (2016). Experimentally increasing titin’s compliance through rbm20 inhibition improves diastolic function in a mouse model of HFpEF. Circulation.

[bib37] Miller MK, Bang ML, Witt CC, Labeit D, Trombitas C, Watanabe K, Granzier H, McElhinny AS, Gregorio CC, Labeit S (2003). The muscle ankyrin repeat proteins: CARP, ankrd2/Arpp and DARP as a family of titin filament-based stress response molecules. Journal of Molecular Biology.

[bib38] Mohamed JS, Lopez MA, Cox GA, Boriek AM (2010). Anisotropic regulation of Ankrd2 gene expression in skeletal muscle by mechanical stretch. FASEB Journal.

[bib39] Molkentin JD (2004). Calcineurin-NFAT signaling regulates the cardiac hypertrophic response in coordination with the MAPKs. Cardiovascular Research.

[bib40] Molkentin JD (2013). Parsing good versus bad signaling pathways in the heart: role of calcineurin-nuclear factor of activated T-cells. Circulation Research.

[bib41] Müller E, Salcan S, Bongardt S, Barbosa DM, Krüger M, Kötter S (2021). E3-ligase knock down revealed differential titin degradation by autopagy and the ubiquitin proteasome system. Scientific Reports.

[bib42] Norrby M, Evertsson K, Fjällström A-K, Svensson A, Tågerud S (2012). Akt (protein kinase B) isoform phosphorylation and signaling downstream of mTOR (mammalian target of rapamycin) in denervated atrophic and hypertrophic mouse skeletal muscle. Journal of Molecular Signaling.

[bib43] Nunes JP, Schoenfeld BJ, Nakamura M, Ribeiro AS, Cunha PM, Cyrino ES (2020). Does stretch training induce muscle hypertrophy in humans? A review of the literature. Clinical Physiology and Functional Imaging.

[bib44] Ottenheijm CAC, van Hees HWH, Heunks LMA, Granzier H (2011). Titin-based mechanosensing and signaling: role in diaphragm atrophy during unloading?. American Journal of Physiology. Lung Cellular and Molecular Physiology.

[bib45] Parker SS, Krantz J, Kwak E-A, Barker NK, Deer CG, Lee NY, Mouneimne G, Langlais PR (2019). Insulin induces microtubule stabilization and regulates the microtubule plus-end tracking protein network in adipocytes. Molecular & Cellular Proteomics.

[bib46] Schiaffino S, Mammucari C (2011). Regulation of skeletal muscle growth by the IGF1-Akt/PKB pathway: insights from genetic models. Skeletal Muscle.

[bib47] Shimoda Y, Matsuo K, Kitamura Y, Ono K, Ueyama T, Matoba S, Yamada H, Wu T, Chen J, Emoto N, Ikeda K (2015). Diabetes-Related Ankyrin Repeat Protein (DARP/Ankrd23) modifies glucose homeostasis by modulating AMPK activity in skeletal muscle. PLOS ONE.

[bib48] Strom J, Bull M, Gohlke J (2024). Titin’s cardiac-specific N2B element is critical to mechanotransduction during volume overload of the heart. Journal of Molecular and Cellular Cardiology.

[bib49] Swist S, Unger A, Li Y (2020). Maintenance of sarcomeric integrity in adult muscle cells crucially depends on Z-disc anchored titin. Nature Communications.

[bib50] Tyanova S, Temu T, Sinitcyn P (2016). The Perseus computational platform for comprehensive analysis of (prote)omics data. Nature Methods.

[bib51] van der Pijl R, Strom J, Conijn S, Lindqvist J, Labeit S, Granzier H, Ottenheijm C (2018). Titin-based mechanosensing modulates muscle hypertrophy. Journal of Cachexia, Sarcopenia and Muscle.

[bib52] van der Pijl RJ, Granzier HL, Ottenheijm CAC (2019). Diaphragm contractile weakness due to reduced mechanical loading: role of titin. American Journal of Physiology. Cell Physiology.

[bib53] van der Pijl RJ, Hudson B, Granzier-Nakajima T, Li F, Knottnerus AM, Smith J, Chung CS, Gotthardt M, Granzier HL, Ottenheijm CAC (2020). Deleting Titin’s C-Terminal PEVK Exons increases passive stiffness, alters splicing, and induces cross-sectional and longitudinal hypertrophy in skeletal muscle. Frontiers in Physiology.

[bib54] van der Pijl RJ, Domenighetti AA, Sheikh F, Ehler E, Ottenheijm CAC, Lange S (2021a). The titin N2B and N2A regions: biomechanical and metabolic signaling hubs in cross-striated muscles. Biophysical Reviews.

[bib55] van der Pijl RJ, van den Berg M, van de Locht M, Shen S, Bogaards SJP, Conijn S, Langlais P, Hooijman PE, Labeit S, Heunks LMA, Granzier H, Ottenheijm CAC (2021b). Muscle ankyrin repeat protein 1 (MARP1) locks titin to the sarcomeric thin filament and is a passive force regulator. The Journal of General Physiology.

[bib56] Voelkel T, Linke WA (2011). Conformation-regulated mechanosensory control via titin domains in cardiac muscle. Pflugers Archiv.

[bib57] Warneke K, Lohmann LH, Lima CD, Hollander K, Konrad A, Zech A, Nakamura M, Wirth K, Keiner M, Behm DG (2023). Physiology of stretch-mediated hypertrophy and strength increases: A narrative review. Sports Medicine.

[bib58] Wette SG, Smith HK, Lamb GD, Murphy RM (2017). Characterization of muscle ankyrin repeat proteins in human skeletal muscle. American Journal of Physiology-Cell Physiology.

[bib59] Wickham H (2019). Github.

[bib60] Yoon MS (2017). mTOR as a key regulator in maintaining skeletal muscle mass. Frontiers in Physiology.

[bib61] Zhong L, Chiusa M, Cadar AG, Lin A, Samaras S, Davidson JM, Lim CC (2015). Targeted inhibition of ANKRD1 disrupts sarcomeric ERK-GATA4 signal transduction and abrogates phenylephrine-induced cardiomyocyte hypertrophy. Cardiovascular Research.

[bib62] Zhou T, Fleming JR, Lange S, Hessel AL, Bogomolovas J, Stronczek C, Grundei D, Ghassemian M, Biju A, Börgeson E, Bullard B, Linke WA, Chen J, Kovermann M, Mayans O (2021). Molecular characterisation of titin N2A and its binding of CARP reveals a titin/actin cross-linking mechanism. Journal of Molecular Biology.

