# Supplementary material for: The titin N2A-MARP signalosome constrains muscle longitudinal hypertrophy in response to stretch
Source: eLife. 2026 Jul 30;14:RP107597. doi: 10.7554/eLife.107597 (PMC13423355; doi:10.7554/eLife.107597)
Supplement: Figure 5—source data 1. [file elife-107597-fig5-data1.pptx]

## Slide 1
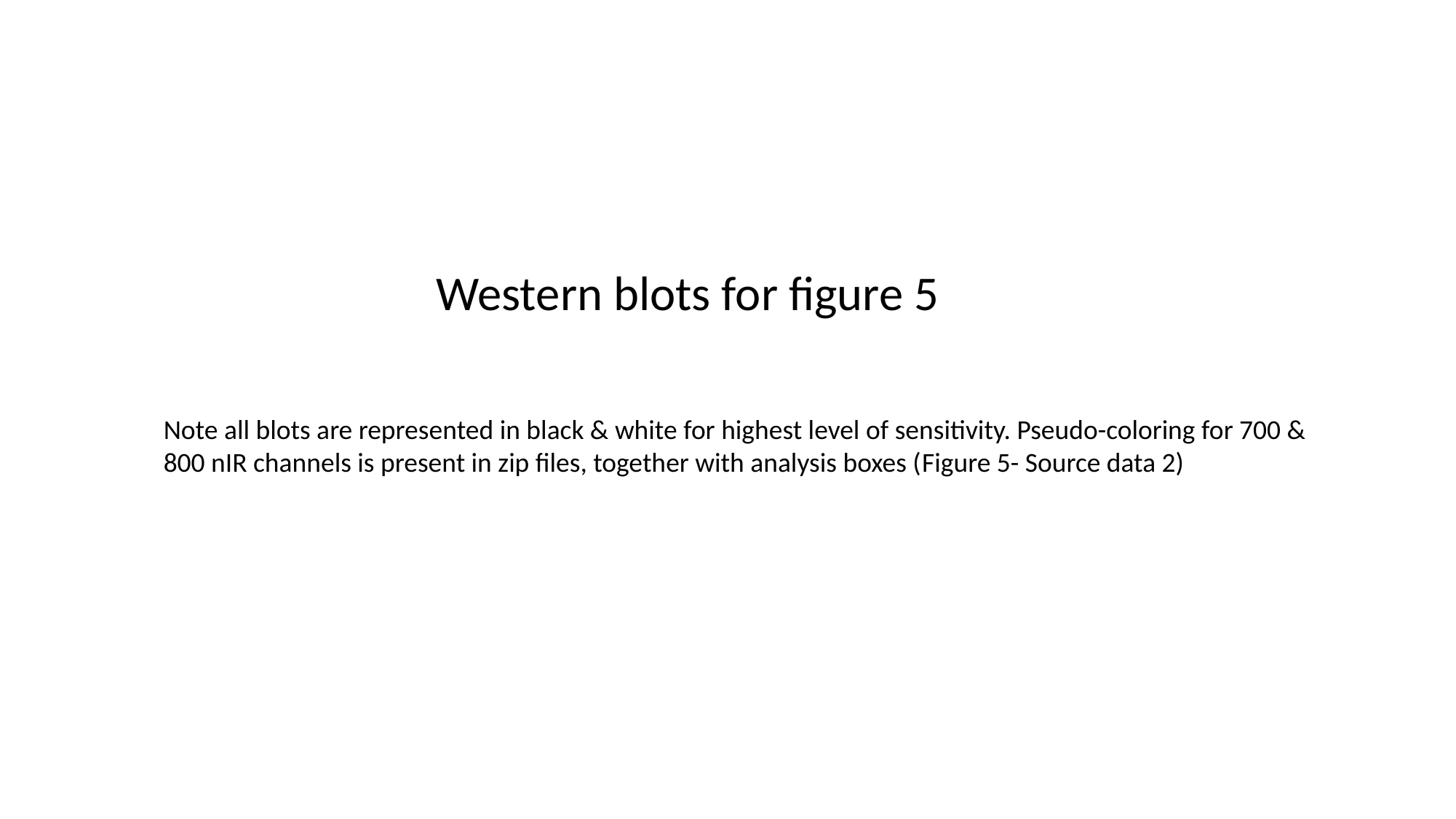

Western blots for figure 5
Note all blots are represented in black & white for highest level of sensitivity. Pseudo-coloring for 700 & 800 nIR channels is present in zip files, together with analysis boxes (Figure 5- Source data 2)

## Slide 2
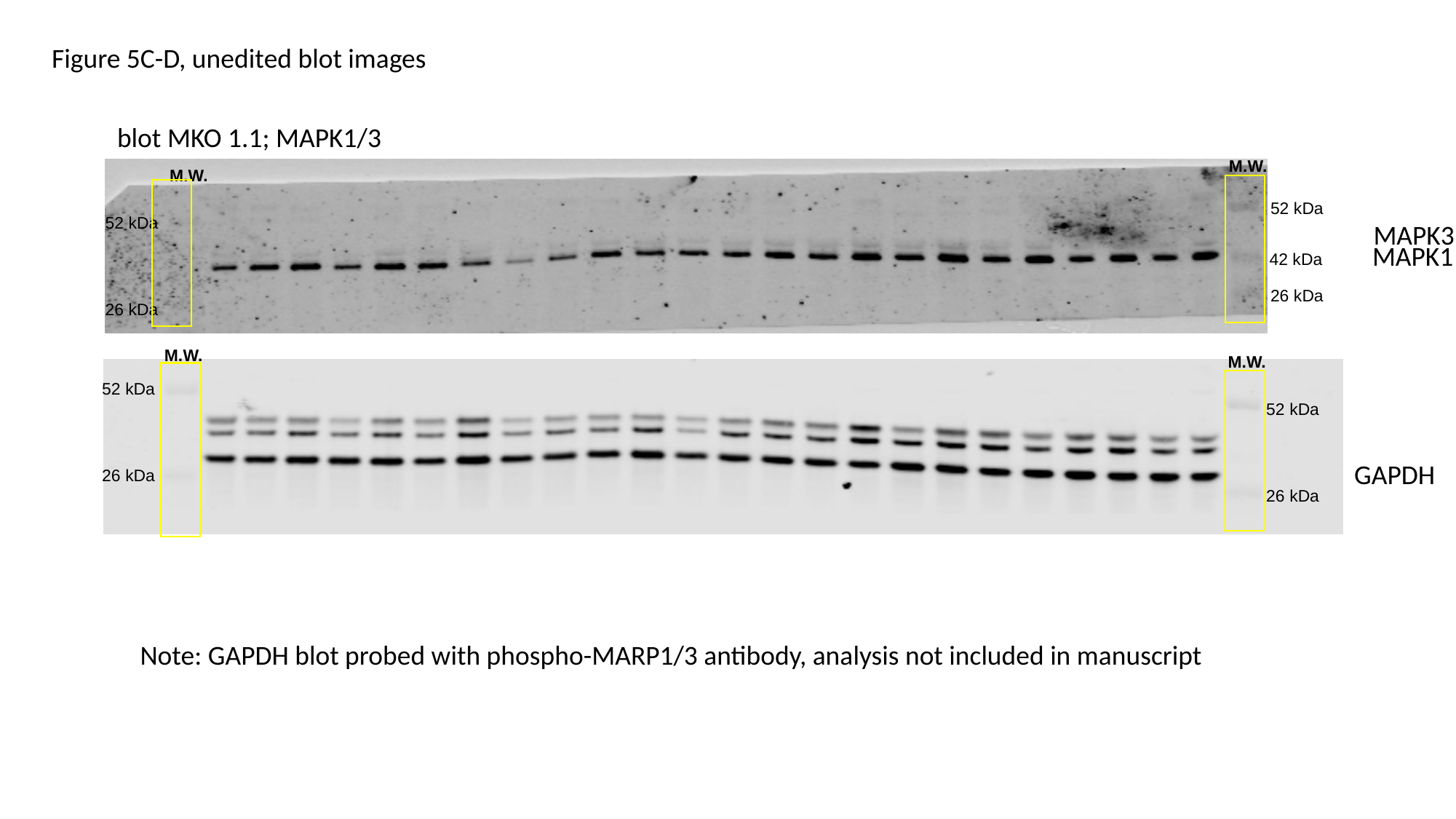

Figure 5C-D, unedited blot images
blot MKO 1.1; MAPK1/3
M.W.
M.W.
52 kDa
52 kDa
MAPK3
MAPK1
42 kDa
26 kDa
26 kDa
M.W.
M.W.
52 kDa
52 kDa
GAPDH
26 kDa
26 kDa
Note: GAPDH blot probed with phospho-MARP1/3 antibody, analysis not included in manuscript

## Slide 3
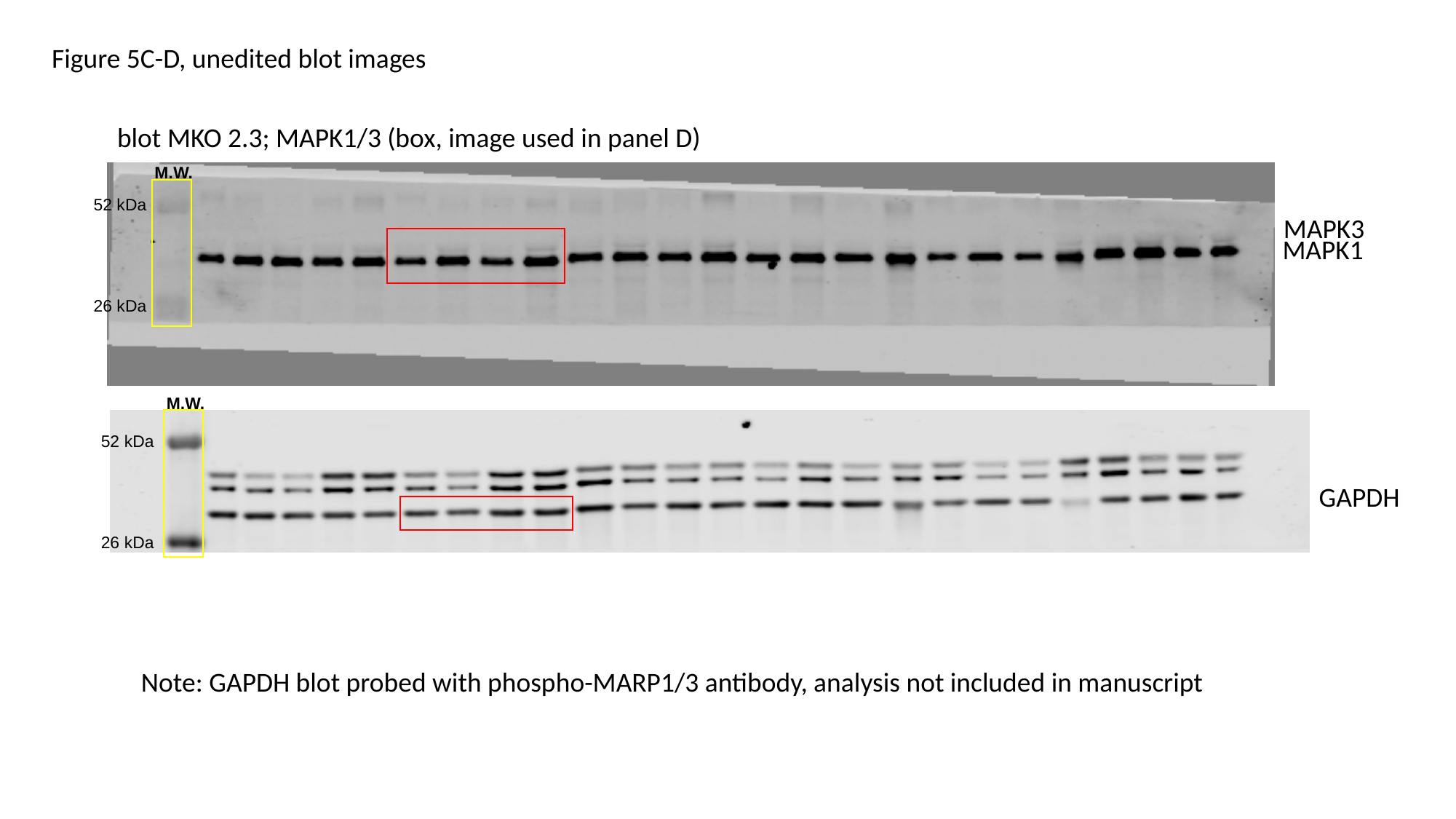

Figure 5C-D, unedited blot images
blot MKO 2.3; MAPK1/3 (box, image used in panel D)
M.W.
52 kDa
MAPK3
MAPK1
26 kDa
M.W.
52 kDa
GAPDH
26 kDa
Note: GAPDH blot probed with phospho-MARP1/3 antibody, analysis not included in manuscript

## Slide 4
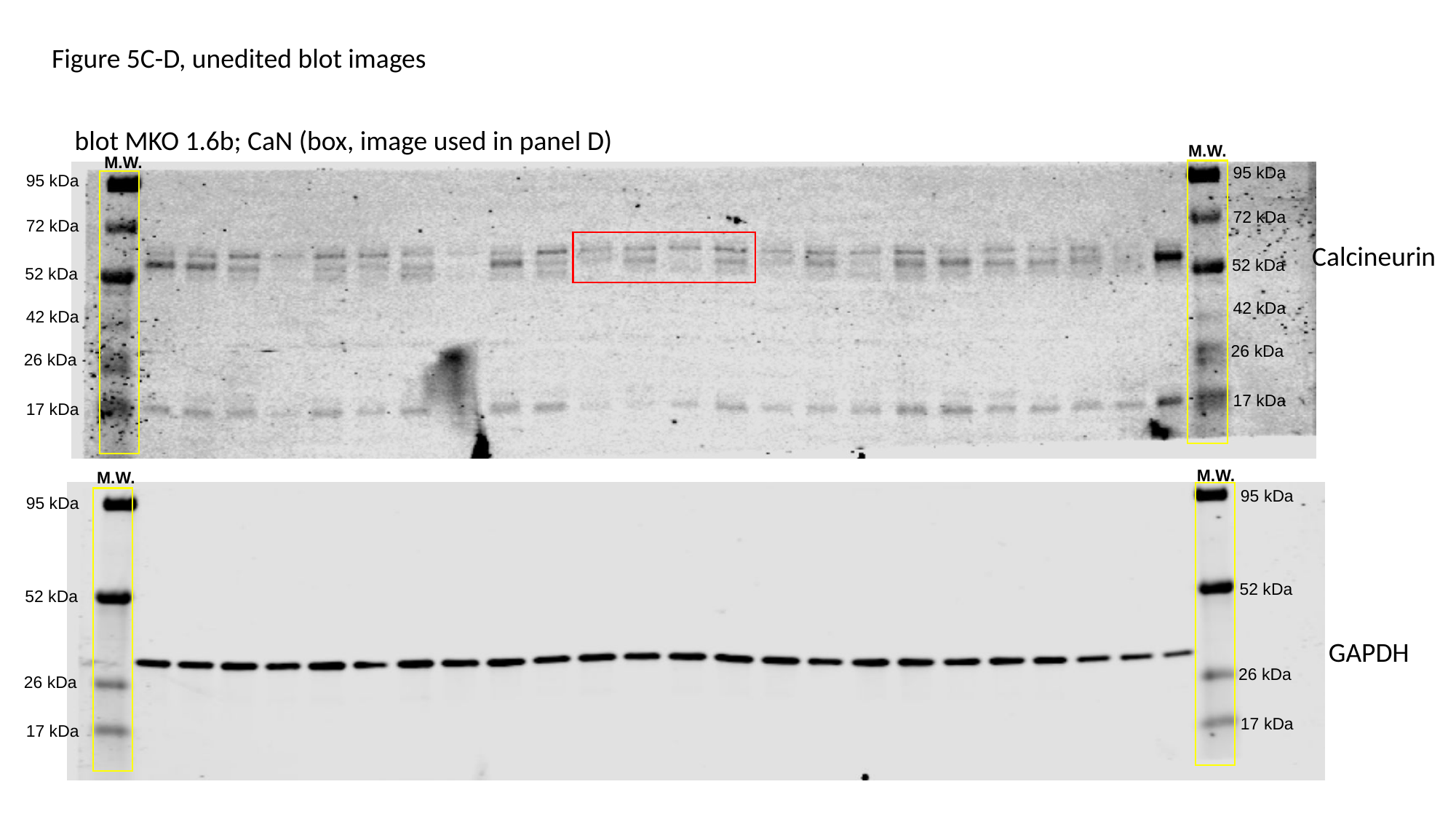

Figure 5C-D, unedited blot images
blot MKO 1.6b; CaN (box, image used in panel D)
M.W.
M.W.
95 kDa
95 kDa
72 kDa
72 kDa
Calcineurin
52 kDa
52 kDa
42 kDa
42 kDa
26 kDa
26 kDa
17 kDa
17 kDa
M.W.
M.W.
95 kDa
95 kDa
52 kDa
52 kDa
GAPDH
26 kDa
26 kDa
17 kDa
17 kDa

## Slide 5
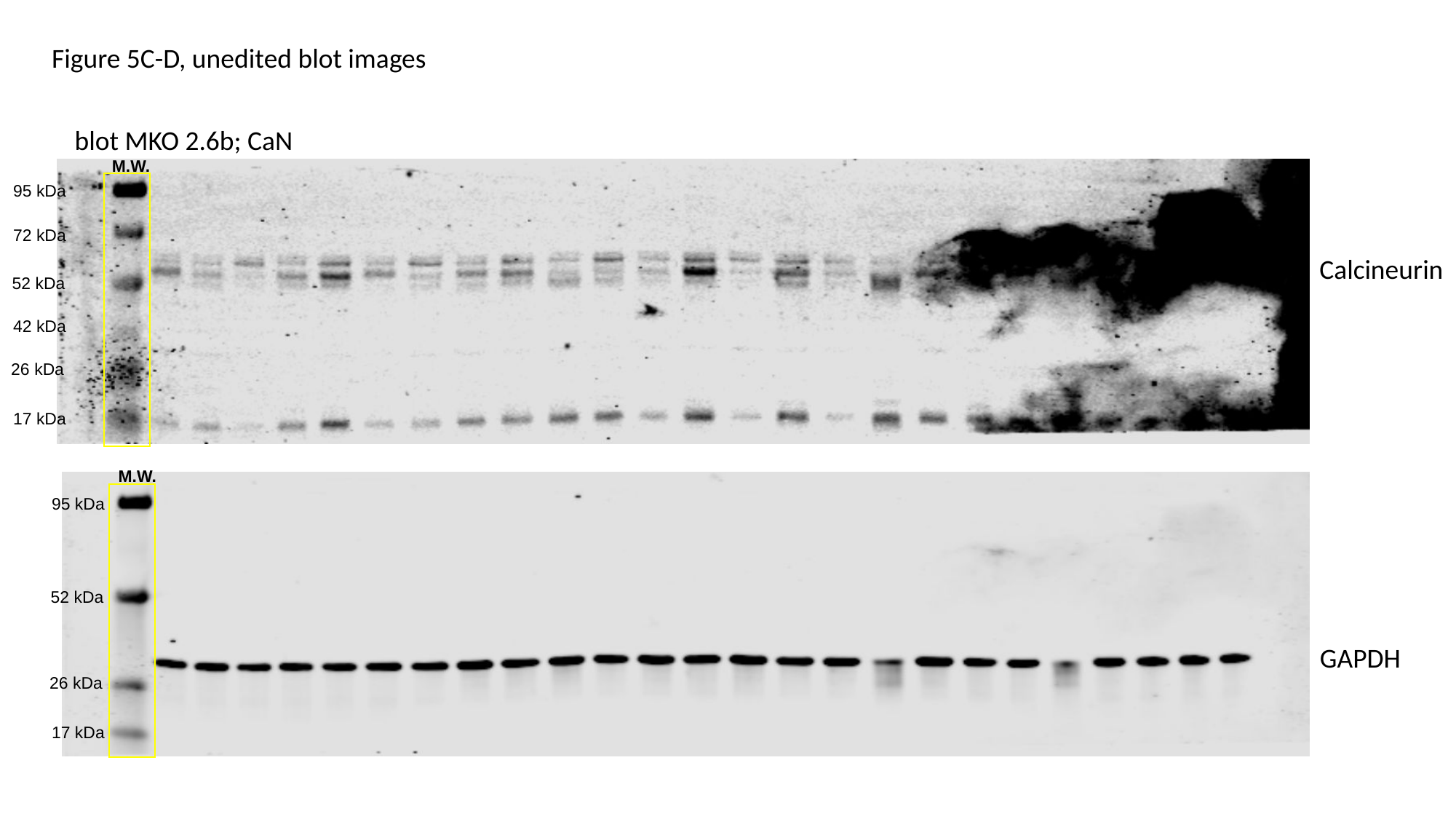

Figure 5C-D, unedited blot images
blot MKO 2.6b; CaN
M.W.
95 kDa
72 kDa
Calcineurin
52 kDa
42 kDa
26 kDa
17 kDa
M.W.
95 kDa
52 kDa
GAPDH
26 kDa
17 kDa

## Slide 6
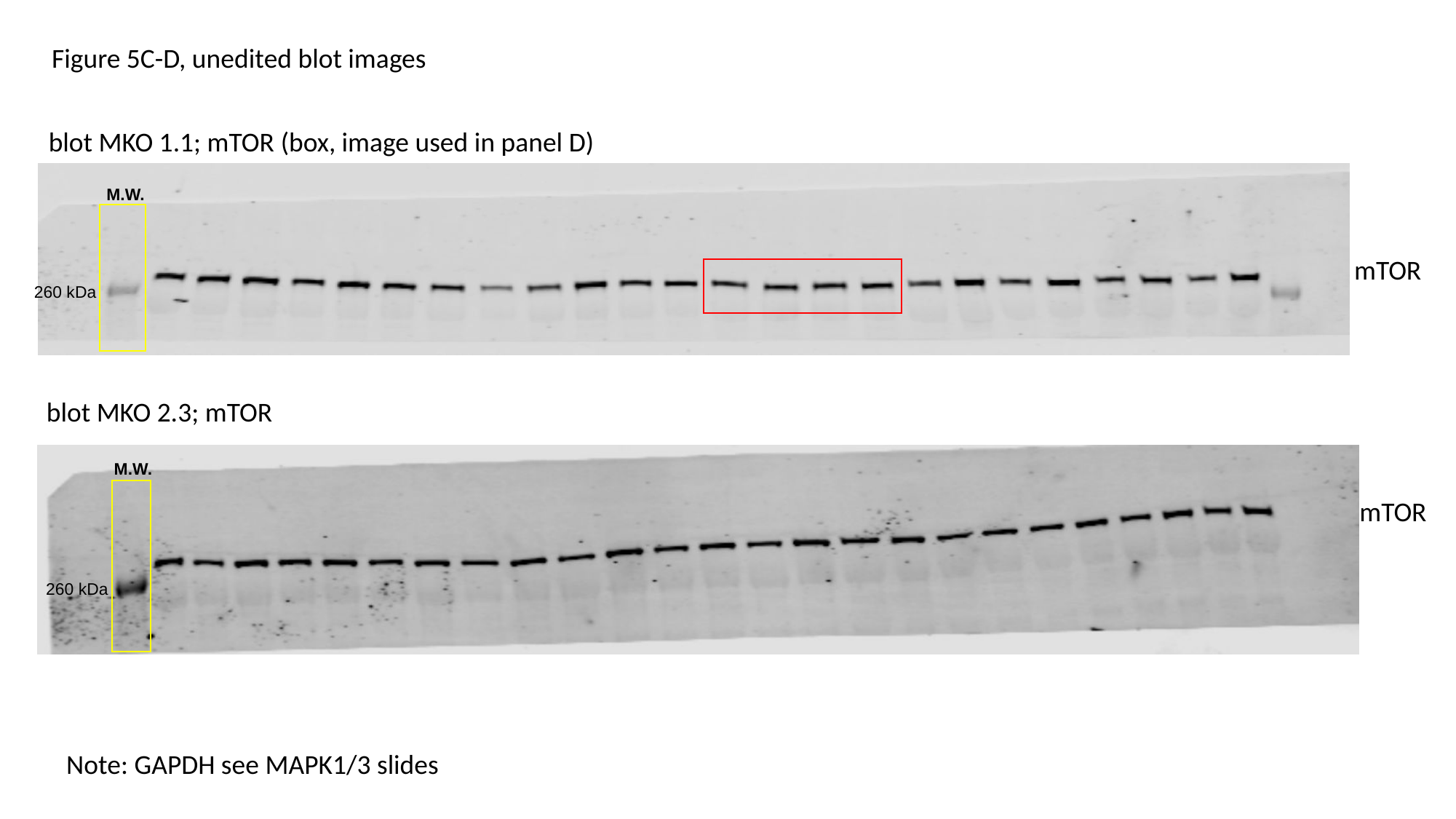

Figure 5C-D, unedited blot images
blot MKO 1.1; mTOR (box, image used in panel D)
M.W.
mTOR
260 kDa
blot MKO 2.3; mTOR
M.W.
mTOR
260 kDa
Note: GAPDH see MAPK1/3 slides

## Slide 7
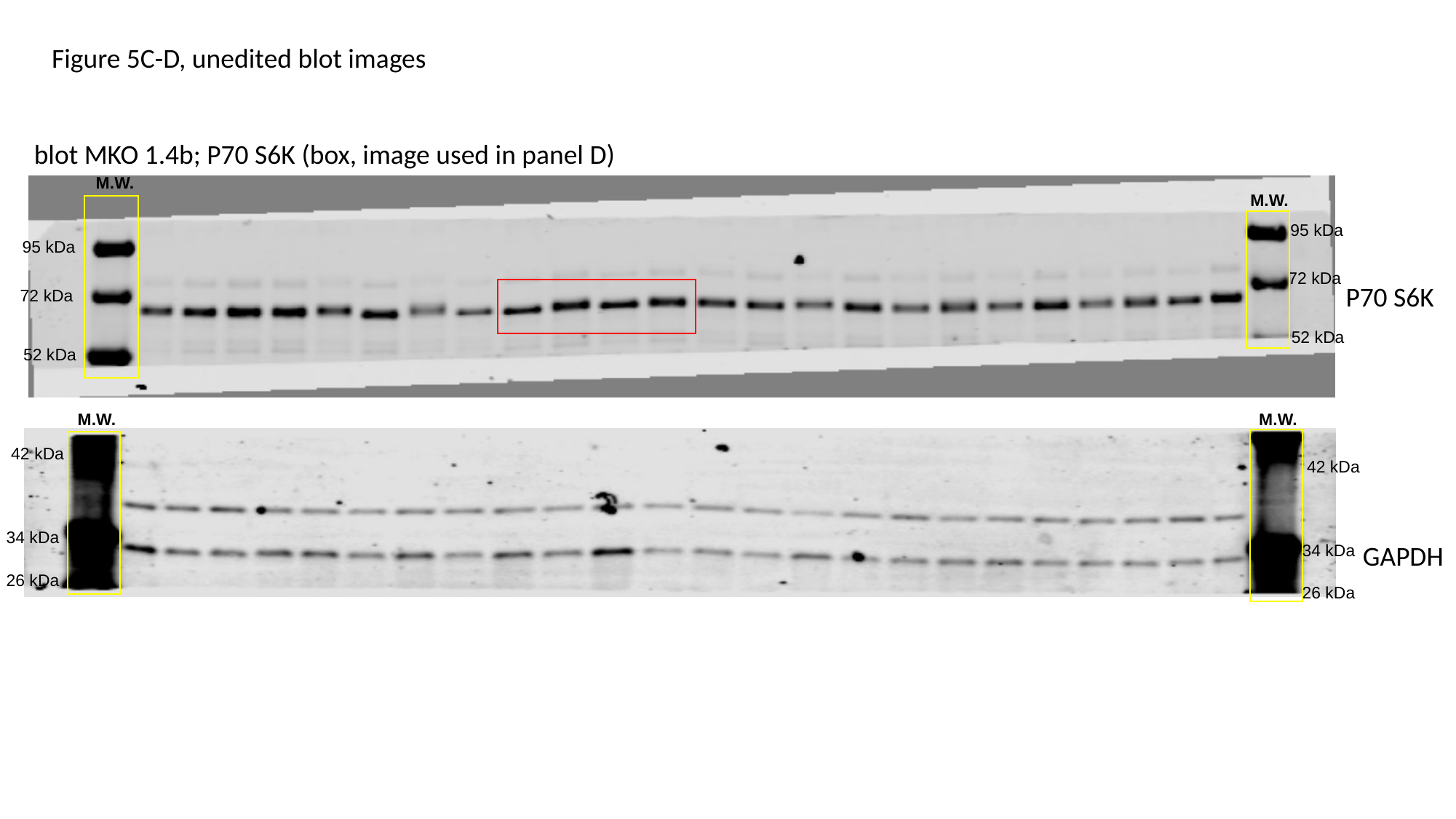

Figure 5C-D, unedited blot images
blot MKO 1.4b; P70 S6K (box, image used in panel D)
M.W.
M.W.
95 kDa
95 kDa
72 kDa
P70 S6K
72 kDa
52 kDa
52 kDa
M.W.
M.W.
42 kDa
42 kDa
34 kDa
GAPDH
34 kDa
26 kDa
26 kDa

## Slide 8
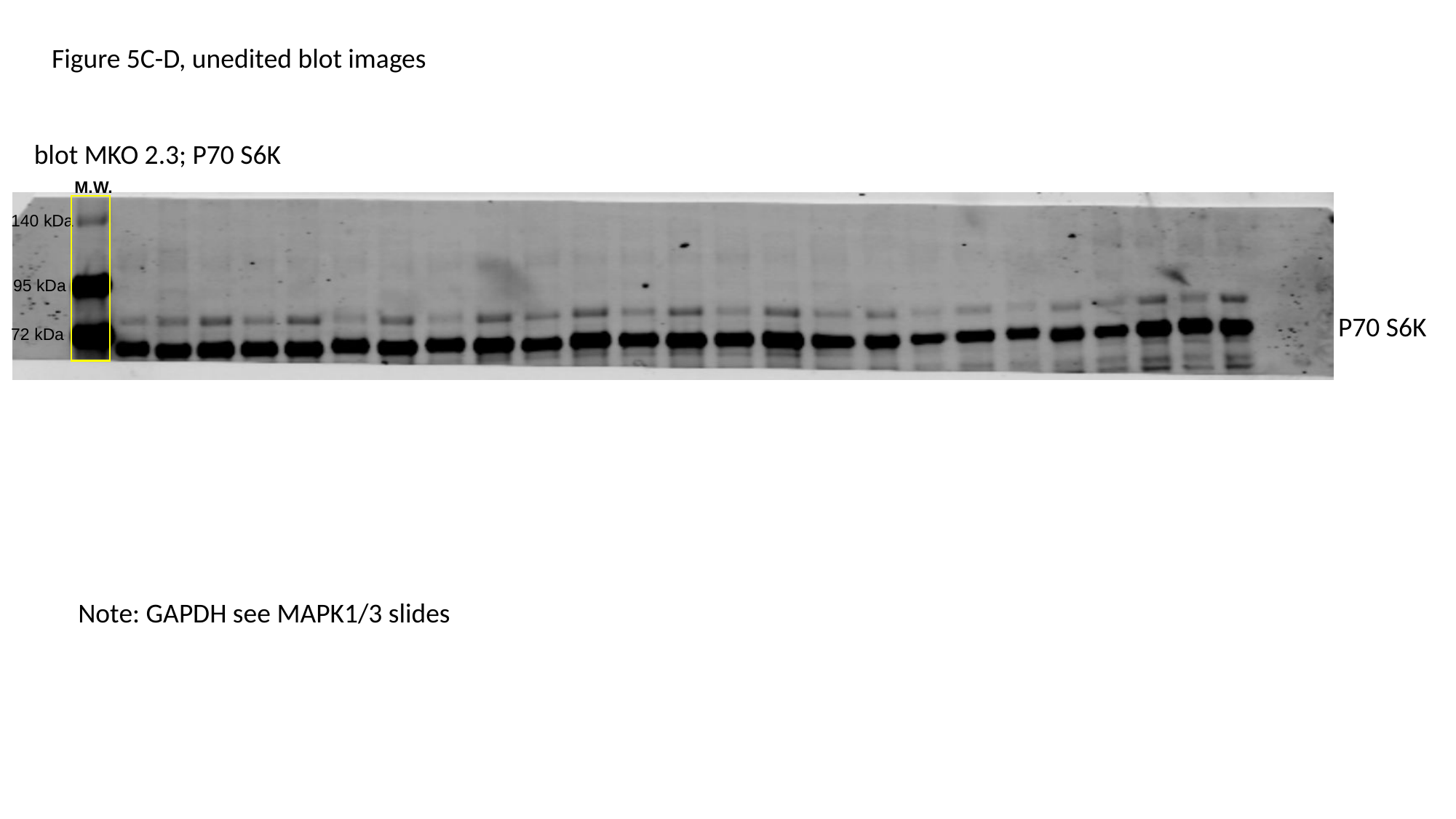

Figure 5C-D, unedited blot images
blot MKO 2.3; P70 S6K
M.W.
140 kDa
95 kDa
P70 S6K
72 kDa
Note: GAPDH see MAPK1/3 slides

## Slide 9
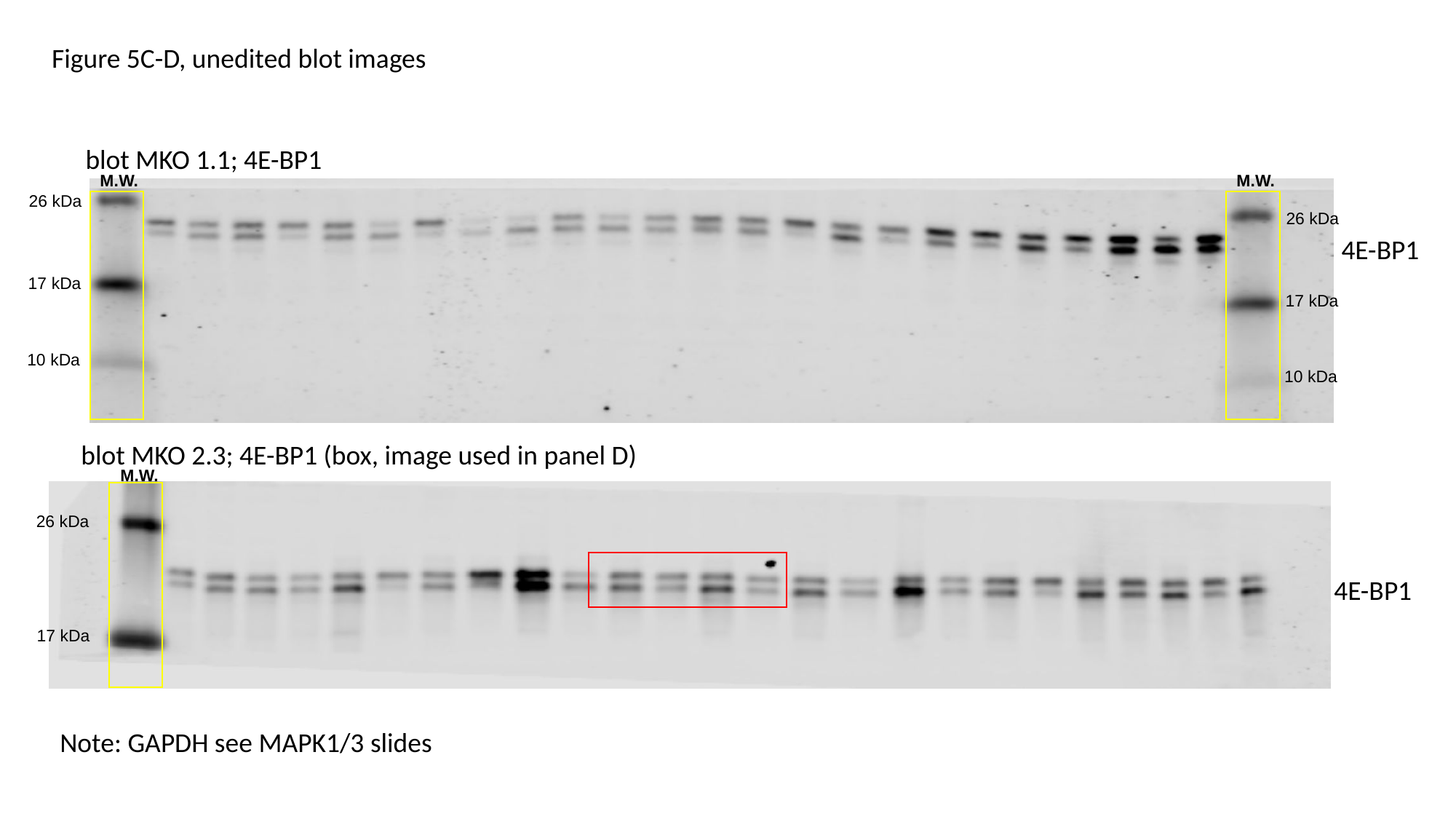

Figure 5C-D, unedited blot images
blot MKO 1.1; 4E-BP1
M.W.
M.W.
26 kDa
26 kDa
4E-BP1
17 kDa
17 kDa
10 kDa
10 kDa
blot MKO 2.3; 4E-BP1 (box, image used in panel D)
M.W.
26 kDa
4E-BP1
17 kDa
Note: GAPDH see MAPK1/3 slides
