# Supplementary material for: The titin N2A-MARP signalosome constrains muscle longitudinal hypertrophy in response to stretch
Source: eLife. 2026 Jul 30;14:RP107597. doi: 10.7554/eLife.107597 (PMC13423355; doi:10.7554/eLife.107597)
Supplement: Peer review history

# Editor report: eLife Assessment

**DOI:** 10.7554/eLife.107597.3.sa0

**Authors:** Benjamin L Parker

**Affiliations:** The University of Melbourne Australia

**Keywords:** Important, Solid

The work by van der Pijl presents **important** findings on the role of titin-associated muscle ankyrin repeat proteins (MARPs) on hypertrophy via mTOR signalling. The study presents rigourous data using in vivo loss-of-function and pharmacological approaches to investigate effects on hypertrophy. While the evidence supporting the role of MARPs on hypertrophy is **solid**, there are limitations. For example, the use of Rapamycin only inhibits some aspects of mTORC1 signalling and the study is limited to analysis of the diaphragm and thus it is not clear if the mechanisms are conserved across other muscle types.

# Referee report: Reviewer #1 (Public review)

**DOI:** 10.7554/eLife.107597.3.sa1

[Editors' note: this version has been assessed by the Reviewing Editor without further input from the original reviewers. The authors have addressed the comments raised in the previous round of review.]

Summary:

In this manuscript, the authors employ diaphragm denervation in rats and mice to study titin-based mechanosensing and longitudinal muscle hypertrophy. By integrating bulk RNA-seq, proteomics, and phosphoproteomics, they map the stretch-responsive signalling landscape, uncovering robust induction of the muscle-ankyrin-repeat proteinsௗ(MARP1-3) together with enhanced phosphorylation of titin's N2A element.

Genetic ablation of MARPs in mice amplifies longitudinal fibre growth and is accompanied by activation of the mTOR pathway, whereas systemic rapamycin treatment suppresses the hypertrophic response, highlighting mTORC1 as a key downstream effector of titin/MARP signalling.

Strengths:

The authors address a clear biological question: "how titin-associated factors translate mechanical stretch into longitudinal fibre growth" using a unique and clinically relevant animal model of diaphragm denervation. Using a comprehensive multiomics approach, the authors identify MARPs as potential mediators of these effects and use a genetic mouse model to provide compelling evidence supporting causality. Additionally, connecting these findings to rapamycin, a drug widely used clinically, further increases the relevance and potential impact of the study.

# Referee report: Reviewer #2 (Public review)

**DOI:** 10.7554/eLife.107597.3.sa2

Summary:

Muscle hypertrophy is a major regulator of human health and performance. Here, van der Pilj and colleagues assess the role of the giant elastic protein, titin, in regulating the longitudinal hypertrophy of diaphragm muscles following denervation. Interestingly, the authors find an early hypertrophic response, with 30% new serial sarcomeres added within 6 days, followed by subsequent muscle atrophy. Using RBM20 mutant mice, which express a more compliant titin, the authors discovered that this longitudinal hypertrophy is mediated via titin mechanosensing. Through an omics approach, it is suggested that the Muscle ankyrin proteins may regulate this approach. Genetic ablation of MARPs 1-3 blocks the hypertrophic response, although single knockouts are more variable, suggesting extensive complementation between these titin binding proteins. Finally, it is found through the administration of rapamycin that the mTOR signalling pathway plays a role in longitudinal hypertrophic growth.

Strengths:

This paper is well written and uses an impressive suite of genetic mouse models to address this interesting question of what drives longitudinal muscle growth.

Weaknesses:

While the findings are of interest, they lack sufficient mechanistic detail in the current state to separate cross-sectional versus longitudinal hypertrophy. The authors have excellent tools such as the RBM20 model to functionally dissect mTOR signalling to these processes. It is also unclear if this process is unique to the diaphragm or is conserved across other muscle groups during eccentric contractions.

# Author comment: Author response

**DOI:** 10.7554/eLife.107597.3.sa3

**Authors:** Robbert van der Pijl, Jochen Gohlke, Joshua Strom, Eva Peters, Shengyi Shen, Stefan Conijn, Zaynab Hourani, Stephan Lange, Ju Chen, Paul Langlais, Siegfried Labeit, Henk L Granzier, Coen Ottenheijm

**Affiliations:** University of Arizona Tucson United States; University of Arizona Tucson United States; University of Arizona Tucson United States; University of Arizona Tucson United States; University of Arizona Tucson United States; University of Arizona Tucson United States; University of Arizona Tucson United States; Aarhus University Aarhus Denmark; University of California, San Diego La Jolla United States; University of Arizona Tucson United States; University of Heidelberg Mannheim Germany; University of Arizona Tucson United States; Amsterdam University Medical Centers Amsterdam Netherlands

The following is the authors’ response to the original reviews.

**eLife Assessment**

The study presents important insights into the regulation of muscle hypertrophy, regulated by Muscle Ankyrin Repeat Proteins (MARPs) and mTOR. The methods are overall solid and complementary, with only minor limitations. Overall, the findings will be of interest for both muscle-biology specialists and the broader mechanobiology community.

We thank the editors for their interest in our manuscript. Below we respond to the reviewer’s comments. Based on these comments we made extensive textual revisions throughout the manuscript, and we added additional analyses to the revised results.

**Reviewer #1 (Public review):**

Summary:

In this manuscript, the authors employ diaphragm denervation in rats and mice to study titin‑based mechanosensing and longitudinal muscle hypertrophy. By integrating bulk RNA‑seq, proteomics, and phosphoproteomics, they map the stretch‑responsive signalling landscape, uncovering robust induction of the muscle‑ankyrin‑repeat proteins (MARP1‑3) together with enhanced phosphorylation of titin's N2A element. Genetic ablation of MARPs in mice amplifies longitudinal fibre growth and is accompanied by activation of the mTOR pathway, whereas systemic rapamycin treatment suppresses the hypertrophic response, highlighting mTORC1 as a key downstream effector of titin/MARP signalling.

Strengths:

The authors address a clear biological question: "how titin‑associated factors translate mechanical stretch into longitudinal fibre growth" using a unique and clinically relevant animal model of diaphragm denervation. Using a comprehensive multiomics approach, the authors identify MARPs as potential mediators of these effects and use a genetic mouse model to provide compelling evidence supporting causality. Additionally, connecting these findings to rapamycin, a drug widely used clinically, further increases the relevance and potential impact of the study.

We thank the reviewer for their kind words and critical review of our manuscript. The roles of the MARP proteins are diverse and form an intriguing target for further study.

Weaknesses:

There are several areas where the manuscript could be substantially improved.

(1) The statistical analysis of multi-omics data needs clarification. Typically, analyses across multiple experimental groups require controlling the false discovery rate (FDR) simultaneously to avoid reporting false-positive findings. It would be very helpful if the authors could specify whether adjusted p-values were calculated using a multi-factorial statistical model (e.g., ~group) or through separate pairwise contrasts.

We agree with the reviewer that the description of the statistical analysis could be improved. We report the q-values in the supplemental data tables to correct for false positive data, the p-values reflect pairwise comparisons. Statistical testing was performed on whole proteomes or phospho-proteomes, making for very stringent testing (please also see reply to reviewer 2, response 5). Unbiased quantitative proteomics functions primarily as a screen, in-solution digestion of muscle proteins yields comparatively few peptides making population adjusted p-value calculation very stringent, suggesting no/few differences in expression. Hence, we compared RNAseq to proteome data to isolate consistently differential proteins. We have revised the method section (lines 745-746) to include clarifications of the FDR analysis.

(2) (A)There are three separate points regarding MARP3 that could be improved. First, the authors report that MARP3-KO mice exhibit smaller increases in muscle mass after diaphragm denervation compared to wild-type mice (a -13% difference), indicating MARP3 likely promotes rather than attenuates hypertrophy. However, the manuscript currently states the opposite (lines 215-216); this interpretation should be revisited. (B) Second, it would be valuable if the authors could provide data showing whether MARP3 transcript or protein levels change response to denervation - if they do not, discussing mechanisms behind the observed phenotype would help clarify the findings. (C) Finally, given that some MARP-KO mice already exhibit baseline differences, employing and reporting the full two-way ANOVA (including genotype × treatment interaction) would allow a direct statistical assessment of whether MARP deficiency modifies the muscle's response to stretch. This analysis would help clearly resolve any existing ambiguity.

(A) Compared to wildtype mice, MARP3 KO mice exhibit baseline diaphragm hypertrophy. This suggests that MARP3 may normally restrain hypertrophy under basal conditions. However, in response to UDD, MARP3 KO mice display an attenuated hypertrophic response, which could be interpreted as MARP3 promoting hypertrophy under stress conditions, as noted by the reviewer. The relationship between MARP3 and metabolism remains incompletely understood, but prior studies indicate that loss of MARP3 enhances glucose tolerance and insulin sensitivity (PMID: 12456686), suggesting that MARP3 may act as a negative regulator of metabolic signaling. Both glucose and insulin can activate the PI3K pathway to promote hypertrophy (PMID: 16679293), which may contribute to the baseline hypertrophy observed in MARP3 KO diaphragms. In addition, MARP3 deficiency has been associated with activation of AMPK signaling (PMID: 26398569). AMPK is a key regulator of metabolic pathways and a well-established inhibitor of hypertrophic signaling, in part through suppression of mTOR activity, and is also responsive to mechanical stimuli (PMID: 18556591). Thus, increased AMPK activity in MARP3 KO mice may limit hypertrophy in response to UDD. Supporting this, our phospho-proteomics data indicate increased activation of the AMPK β-subunit following UDD, suggesting a potential role for AMPK signaling in stretch-induced hypertrophy. Based on these considerations, we have removed the statement that MARP3 attenuates hypertrophy and instead incorporated the potential role of AMPK signaling into the Discussion (lines 354–355). While the present study focuses on the triple MARP KO model, future work will examine the specific contributions of individual MARP proteins to muscle hypertrophy.

(B) MARP3 (Ankrd23) upregulation at the RNA level was detected by RNA-seq in rat diaphragm following both UDD and BDD (Supplemental Tables 1 and 2). This is consistent with our prior findings in mice, where western blot analysis showed increased MARP3 protein expression following UDD (PMID: 29978560). We note that reliable detection of MARP3 protein remains technically challenging due to limited availability of specific antibodies.

(C) We agree with the reviewer and have added the results of the two-way ANOVA to the figures (see updated Figure 4). The three MARP proteins exhibit differential effects on diaphragm hypertrophy, supporting their role as modulators of stretch-induced hypertrophy.

(3) The current presentation of multi-omics data is somewhat difficult to follow, making it challenging to determine whether observed changes occur at the transcript or protein level due to inconsistent gene/protein naming and capitalization (e.g., proper forms are mTOR, p70 S6K, 4E-BP1). Clearly organizing and presenting transcript and protein-level changes side-by-side, especially for key molecules discussed in later experiments, would make the data more accessible and provide clearer insights into the biology of titin-mediated mechanosensing.

We agree with the reviewer that naming conventions between gene and protein can be hard to follow. We kept the names for titin-associated proteins as some have multiple protein names and the most common names is shown here. However, we made the suggested changes for the mTOR related proteins (for example, see figure 5).

(4) The current analysis relies on total protein measurements downstream of mTOR, yet mTOR's primary mode of action is to change phosphorylation status. Because the authors have already generated a phosphoproteomic dataset, it would be very helpful to report - or at least comment on - whether known mTOR target phosphosites were detected and how they respond to denervation and rapamycin. Including even a brief summary of canonical sites such as S6K1 Thr389 or 4E - BP1 Thr37/46 would make the link between mTOR activity and hypertrophy much clearer.

We agree with the reviewer that the mTOR data requires more work to ascertain its function in regulating hypertrophy following UDD. We investigated S6K1 Thr389 or 4E BP1 Thr37/46 in both the phosphoproteomic dataset and by western blot. These sites do not appear in phosphoproteome mass spectrometry (supplemental data table 13) and 4E BP1 Thr37/46 was unchanged by western blot (not shown). The S6K1 Thr389 antibody was aspecific in our hands, but Norrby et al (PMID: 22657251) saw increased levels by 6-days UDD. Hence the mTOR aspect of this study is quite complex, suggesting mTOR plays a major role in UDD hypertrophy, but potentially through an alternative activation pathway from what is classically described for muscle hypertrophy. We are investigating the mTOR mechanism further focusing on mTOR’s role in regulating longitudinal hypertrophy with potential connection to titin signaling and hope to publish this in the next few years. We revised the discussion to include canonical mTOR activation in hypertrophy, please see lines 388-392.

(5) Finally, since rapamycin blocks only a subset of mTOR signalling, a brief discussion that distinguishes rapamycin‑sensitive from rapamycin‑insensitive pathways would be valuable. Clarifying whether diaphragm stretch relies exclusively on the sensitive branch or also engages the resistant branch would place the results in a broader mTOR context and deepen the mechanistic narrative.

We agree with the reviewer that distinguishing between rapamycin-sensitive and -insensitive mTOR signaling adds useful context to the interpretation of stretch-induced hypertrophy. Rapamycin primarily inhibits mTORC1, whereas mTORC2 is generally considered rapamycin-insensitive, although prolonged or high-dose exposure can also affect mTORC2 activity. Our data indicate that UDD induces a form of hypertrophy that is sensitive to rapamycin, supporting a prominent role for mTORC1 in this process. However, we cannot exclude the possibility that rapamycin-insensitive pathways, including mTORC2 signaling, also contribute. Notably, denervation itself may influence mTORC2 activity, which could complicate the distinction between stretch- and denervation-mediated signaling. Given these considerations, we have added a brief discussion to acknowledge potential contributions of rapamycin-insensitive mTOR signaling (lines 379-384). A more comprehensive dissection of mTORC1 versus mTORC2 signaling in this context will require targeted approaches and falls beyond the scope of the present study.

**Reviewer #1 (Recommendations for the authors):**

Minor comments:

(6) The manuscript notes that KEGG analysis "confirmed" the GO‑term findings. Because KEGG pathways and GO terms describe different types of biological information, it might be clearer simply to present them as complementary lines of evidence rather than one validating the other.

We agree and modified the text accordingly. “Concurrently, KEGG PATHWAY database searches (Supplemental data Table 6) indicated that the DEG’s are involved in muscle remodeling.” See lines 166-169.

(7) Figure 2's legend mentions a two‑way ANOVA, but the specific factors tested are not specified. Listing those two factors would help readers interpret the statistics more easily.

The two-way ANOVA refers to the violin plot in figure 2E and tests the difference of the 2 surgical modalities sham vs UDD and sham vs BDD. Sham groups were combined in the graphs for easy comparison. We clarified the text of figure legend 2.

(8) The Methods briefly describe phosphopeptide enrichment, but additional details on the criteria for site identification - such as the localisation algorithm, probability cut‑off, and FDR thresholds - would make the phosphoproteomics section more transparent and reproducible.

Please see the updated method section, lines 756-765

**Reviewer #2 (Public review):**

Summary:

Muscle hypertrophy is a major regulator of human health and performance. Here, van der Pilj and colleagues assess the role of the giant elastic protein, titin, in regulating the longitudinal hypertrophy of diaphragm muscles following denervation. Interestingly, the authors find an early hypertrophic response, with 30% new serial sarcomeres added within 6 days, followed by subsequent muscle atrophy. Using RBM20 mutant mice, which express a more compliant titin, the authors discovered that this longitudinal hypertrophy is mediated via titin mechanosensing. Through an omics approach, it is suggested that the Muscle ankyrin proteins may regulate this approach. Genetic ablation of MARPs 1-3 blocks the hypertrophic response, although single knockouts are more variable, suggesting extensive complementation between these titin binding proteins. Finally, it is found through the administration of rapamycin that the mTOR signalling pathway plays a role in longitudinal hypertrophic growth.

Strengths:

This paper is well written and uses an impressive suite of genetic mouse models to address this interesting question of what drives longitudinal muscle growth.

We appreciate the reviewer’s kind words on our manuscript and their critical review of our work. A potential separate mechanism governing cross-sectional versus longitudinal hypertrophy is of great interest and something we aim to address in future manuscripts.

Weaknesses:

While the findings are of interest, they lack sufficient mechanistic detail in the current state to separate cross-sectional versus longitudinal hypertrophy. The authors have excellent tools such as the RBM20 model to functionally dissect mTOR signalling to these processes. It is also unclear if this process is unique to the diaphragm or is conserved across other muscle groups during eccentric contractions.

**Reviewer #2 (Recommendations for the authors):**

(1) Cross-sectional hypertrophy characterization: The paper emphasizes longitudinal hypertrophy but does not quantify the contribution of radial (cross-sectional) hypertrophy to the total mass increase. Given that the denervated costal diaphragm shows ~50% increase in mass (Figure 1B) but there is only ~30% fiber lengthening, it is important to determine the proportion attributable to fiber diameter changes. Histological analysis of muscle fiber cross-sectional area would clarify the relative contributions of longitudinal versus radial hypertrophy to the overall mass phenotype.

We agree with the reviewer that radial hypertrophy is an important mechanism for muscle weight gain in UDD. In previous work we characterized both the radial and longitudinal hypertrophy response in 6-day UDD and found that ~20% of the mass gain seen in UDD is radial hypertrophy (PMID: 29978560). We reference this paper in the discussion section, line 277-278. Doing a full histological work-up of UDD diaphragm would be interesting but falls outside the scope of this manuscript. Our focus was to characterize longitudinal hypertrophy by addition of sarcomeres in series and provide insight into titin’s role in regulating longitudinal hypertrophy. We hope that the reviewer agrees with this approach.

(2) Titin isoform expression analysis: At line 103, the authors propose that longitudinal hypertrophy reduces strain on titin by decreasing fractional sarcomere extension. However, this hypothesis does not exclude the possibility of isoform switching to a less elastic titin variant, which may compensate for changes in mechanical stress. The RNA-sequencing data should be analyzed for titin exon usage patterns between sham and UDD to determine whether changes in isoform composition (e.g., PEVK region splicing) accompany longitudinal hypertrophy. If isoform switching occurs, this represents an alternative or complementary mechanism to sarcomere addition.

We analyzed titin exon usage in rat following both UDD and BDD. Increases in sarcomeres in series associated with UDD show modest changes in titin exon usage, though not significant by population adjusted p-values. The denervation effect of BDD did show changes in splicing, indicating lower inclusion of PEVK encoding exons, suggesting a stiffening of the titin molecules. Stiffening of titin molecules might be protective for the fully paralyzed diaphragm and preserve muscle mass. This would align with our prior publication (PMID: 29978560) which showed that stiffer titin generated more radial hypertrophy in response to UDD. In response to the reviewer’s comment, we added the splicing data to the supplemental data as new figure 2 and briefly address titin splicing in the results section, see lines 121-125.

(3) The comparison of 3-day unilateral diaphragm denervation (UDD) and bilateral diaphragm denervation (BDD) in rats (Figure 1D-E) is used to argue that hypertrophic signaling is stretch-dependent rather than denervation-dependent. However, this interpretation requires clarification. In mice, hypertrophy is detectable as early as 1 day post-UDD, whereas the 3-day BDD protocol may drive an accelerated hypertrophic-to-atrophic remodelling process given the severity of the model. Moreover, longitudinal and global muscle hypertrophy may operate through distinct mechanisms: denervation could suppress longitudinal hypertrophy through a separate pathway while promoting or delaying cross-sectional hypertrophy. The authors should acknowledge that the current evidence does not fully exclude denervation-dependent mechanisms and should consider extended BDD time points or additional mechanistic studies to clarify this distinction.

UDD and BDD are both denervation models and hypertrophy occurs in the denervated costal of UDD operated animals. Stretch is thus the mechanical difference between UDD and BDD and thus the trigger for hypertrophy signaling. At the denervation signaling level both models should in principle be comparable and are unlikely to play different roles between UDD and BDD, except that UDD also induces a more potent hypertrophy signaling profile on top of the atrophy program. That said, BDD is a more severe model and respiration rate is depressed compared to UDD where respiration rate is elevated. BDD rats also engage in abdominal breathing, which mildly stretches the diaphragm. Hypoxia is likely to play a stronger role in BDD than UDD and could thus further enhance the atrophy profile of BDD. We agree with the reviewer that more work is needed to elucidate the BDD remodeling response, however UDD induced stretch is the main driver of longitudinal hypertrophy. In response to the reviewer’s comment, we have added clarifying text to the discussion, lines 286-292.

The potential for there being two independent mechanisms for both radial and longitudinal hypertrophy is of great interest to us. We foresee that dissecting out these differences will require a cell culture-based approach and will aid in avoiding the complexity of overlapping denervation and hypertrophy signals as seen in this manuscript.

(4) Characterization of RBM20 models: The RBM20 experiments rely on the assumption that increased titin compliance reduces stretch sensitivity. However, the paper provides minimal baseline characterization of the diaphragms. Specifically: (a) What are the sarcomere lengths in RBM20-deficient diaphragms at rest and under stretch? (b) How does the passive force-length relationship differ between wildtype and RBM20-deficient diaphragm muscles? and (c) Would RBM20-deficient muscles, despite having longer sarcomeres at baseline, actually experience sufficient strain to activate mechanosensing? These data are necessary to interpret why RBM20-deficient mice show attenuated mass gain rather than none (as in BDD) during UDD (Supplemental Figure 2A-C). Additionally, what would the authors hypothesize would happen if rapamycin were used in RMB20 UDD models? It appears to be an attractive experimental approach to separate potential mTOR contributions to longitudinal versus cross-sectional hypertrophy.

We agree with the reviewer that more work is needed on Rbm20 deficient mice and rats to elucidate their response to stretch. Part of this characterization has previously been published (PMID: 29978560) and Rbm20 splice-deficient mice have reduced passive stiffness in the diaphragm and show a robust mechanosensing response to UDD. Rbm20 splice-deficient mice also show a similar increase in longitudinal hypertrophy, but a blunted radial hypertrophy in response to 6-days UDD. The main reason for not expanding on these mice/rats further was the added complexity of Rbm20 splicing multiple targets that could affect hypertrophy signaling, for example LDB3 (ZASP) and FLNC (Filamin C) are both associated with hypertrophic cardiomyopathy. Hence for the purpose of this manuscript we showed mice and rats having a similar response to UDD, hypertrophy wise, and that titin stiffness (reduced in Rbm20-deficient animals) affects hypertrophy at the diaphragm mass level.

Testing rapamycin on Rbm20-deficient animals could be interesting, however the complexities of also changing splicing of non-titin targets will make interpretation of mTOR signaling difficult. Perhaps an alternative approach would be to generate a titin mouse model with more compliant titin (e.g. increase the size of the PEVK segment), a model we are considering for future studies. TtnΔ112-158 mice, deleting a large portion of the PEVK region (PMID: 30565562) show increases in sarcomere number. We would expect a model with more PEVK to thus show a reduction in the number of sarcomeres in series. We discuss the role of titin stiffness in the discussion and how titin stiffness ties to longitudinal hypertrophy, please see lines 302-314.

(5) Statistical analysis and multiple hypothesis correction: The proteomic analyses appear to employ a nominal p-value threshold (p < 0.05) without correction for multiple comparisons or false discovery rate (FDR) control. This is particularly concerning given the large number of comparisons. For example, the authors report 142 titin phosphorylation sites significantly different between sham and UDD at p < 0.05 (approximately 20% of ~700 identified sites). However, with proper FDR correction (adjusted p < 0.05), only 14 sites remain significant - a 90% reduction. This discrepancy is critical for the discussion on titin N2A phosphorylation sites pS9459 and pS9520, where only pS9520 achieves statistical significance after FDR adjustment. The authors should justify their choice of statistical thresholds and reanalyze key findings using FDR-corrected p-values. Additionally, the phosphoproteomics dataset should be screened for duplicate phosphosite identifications to ensure each site is counted only once.

Reviewer 1 has voiced similar concerns, and we have thus expanded the methodology to explain the statistical tests used to analyze the data and the process of establishing Z-scores of isobaric peptides for the same phospho-sites (see lines 756-765). Our statistical analysis covers all detected peptides, when we only analyze the titin peptides: pS9459 is only significant in t-test, likely due to large variation in isobaric peptides. pS9520 is significant in both independent t-test and FDR. We changed figure 3D to show the fold change instead of the previous Z-score for more intuitive interpretation.

Minor comments:

(6) Line 52: "thesarcomeres" should read "the sarcomeres".

A space has been added, please see line 52.

(7) Line 52: "half-sarcomer" should read "half-sarcomere"

Spelling has been corrected, please see line 52.

(8) Figure clarity: Figure 1 (B-C) presents mouse data, while Figure 1 (D-E) presents rat data. This distinction should be clearly labeled in the figure legend or on the axes to prevent misinterpretation, particularly for readers unfamiliar with the experimental design.

We added the species to the y-axis of revised figure 1B-E and added additional clarification in the figure legend.

(9) Supplementary tables: When reporting statistical comparisons in the supplementary tables, please consider including the directionality of the statistical tests (e.g., which group was higher or lower) alongside p-values. This will facilitate interpretation without requiring reference to the main text figures.

We agree with the reviewer and added statistical direction as a new column next to the p-values, please see the revised supplemental tables.

(10) Given the interesting divergent findings in MARPtKO versus single knockouts, it would be interesting to assess by immunofluorescence the association of each MARP with the N2A region of titin following UDD.

We agree with the reviewer that localization is important. Miller et al (PMID: 14583192) previously localized MARP1-3 to the N2A segment by immuno-EM and our work previously localized MARP1 to N2A using SR-SIM (PMID: 29978560). We will further investigate MARPs binding to the N2A region in an upcoming study that we intend to publish soon.
